# Phosphorylation of *Xenopus* p31^comet^ potentiates mitotic checkpoint exit

**DOI:** 10.1080/15384101.2015.1033590

**Published:** 2015-04-18

**Authors:** Min Mo, Alexei Arnaoutov, Mary Dasso

**Affiliations:** Laboratory of Gene Regulation and Development; National Institute of Child Health and Human Development; National institutes of Health; Bethesda, MD USA

**Keywords:** inhibitor of nuclear factor κ-B kinase-β, (IKK-β), Mad2, p31^comet^, Spindle assembly checkpoint, Xenopus

## Abstract

p31^comet^ plays an important role in spindle assembly checkpoint (SAC) silencing. However, how p31^comet^'s activity is regulated remains unclear. Here we show that the timing of M-phase exit in *Xenopus* egg extracts (XEEs) depends upon SAC activity, even under conditions that are permissive for spindle assembly. p31^comet^ antagonizes the SAC, promoting XEE progression into anaphase after spindles are fully formed. We further show that mitotic p31^comet^ phosphorylation by Inhibitor of nuclear factor κ-B kinase-β (IKK-β) enhances this role in SAC silencing. Together, our findings implicate IKK-β in the control of anaphase timing in XEE through p31^comet^ activation and SAC downregulation.

## Abbreviations

SAC: spindle assembly checkpoint
XEEs: Xenopus egg extracts
IKK-: β Inhibitor of nuclear factor κ-B kinase-β
KT: kinetochore
MT: microtubule
NEBD: nuclear envelope breakdown
APC/C: anaphase promoting complex/cyclosome
CSF: cytostatic factor
DSN: demembranated sperm nuclei
TPCA: [5-(p-fluorophenyl)-2-ureido] thiophene-3-carboxamide
MCC: itotic checkpoint complex.

## Introduction

The spindle assembly checkpoint (SAC) ensures faithful separation of mitotic sister chromatids by monitoring interactions between kinetochores (KTs) and microtubules (MTs) (reviewed in^1–3^). The SAC controls the anaphase promoting complex/cyclosome (APC/C), a ubiquitin ligase that is crucial for mitotic exit. SAC activation causes sequestration of an APC/C activator, Cdc20, within an inhibitory complex called the mitotic checkpoint complex (MCC). The MCC is composed of Cdc20 and SAC proteins Mad2, BubR1, Bub3. The Mad2 protein exists in 2 states, the inactive open state (o-Mad2) and the active closed state (c-Mad2); c-Mad2 is incorporated into the MCC complex. Conversion of o-Mad2 to c-Mad2 occurs at a “catalytic platform” complex on unattached KTs, comprised of c-Mad2 and Mad1.

Mad1 and Mad2 are not essential in budding yeast unless KT MTs are disrupted,^4^ suggesting that the SAC is not required for spindle structure or chromosome segregation *per se*. However, Mad1 and Mad2 are essential for correct chromosome segregation and organismal viability in metazoans,^5,6^ indicating that the SAC is required in higher eukaryotes even without external perturbation of spindle assembly. This difference reflects the fact that yeast KTs are constitutively MT bound throughout the cell cycle, whereas organisms that undergo open mitosis re-establish KT-MT attachments after nuclear envelope breakdown (NEBD) in each cell division. Unattached KTs transiently invoke the SAC during the interval between NEBD and MT attachment, and subsequent SAC silencing helps to determine the timing of anaphase onset.^6^

SAC silencing is not fully understood, although it clearly involves a number of crucial events, including both inactivation of the catalytic platform at KTs and disassembly of existing MCC.^1^ p31^comet^ is a protein found in higher eukaryotes that plays an important role in SAC silencing.^7^ p31^comet^ over-expression in tissue culture cells overrides the SAC, while p31^comet^ knockdown delays anaphase onset.^8,9^ p31^comet^ is structurally related to Mad2^10^ and it binds c-Mad2 in a manner that is essential for its role in SAC silencing.^8,11^ Two mechanisms have been suggested for p31^comet^ activity: First, p31^comet^ has been proposed to disrupt soluble MCC. This model is supported by the capacity of p31^comet^ to bind c-Mad2 within the MCC *in vitro* and thereby disrupt that complex.^12^ Second, p31^comet^ associates with unattached KTs through direct binding to c-Mad2.^8,11^ p31^comet^ binding to c-Mad2 occludes o-Mad2 binding,^10^ and a “capping” model has been proposed wherein p31^comet^ blocks MCC generation by inhibition of the Mad1/c-Mad2 catalytic platform.^13^

Xenopus egg extracts (XEEs) are a convenient system for *ex vivo* studies on mitosis. CSF-XEEs preserve the meiotic arrest of the frog egg by cytostatic factor (CSF).^14^ CSF-XEEs form spindles after addition of demembranated sperm nuclei (DSN). Upon the addition of CaCl_2_, which mimics fertilization of intact eggs, CSF is lost, spindles are disassembled and CSF-XEEs proceeds into interphase. XEE can recapitulate cell cycle checkpoints, including the SAC. For example, the SAC becomes activated by unattached KTs in CSF-XEEs containing DSN and the MT-depolymerizing agent nocodazole, preventing anaphase onset even after CaCl_2_ addition and CSF degradation. Cycling XEE are produced through an alternative protocol, so that they mimic the cell cycle of the fertilized egg, spontaneously alternating between interphase and mitosis.^14^ Like CSF-XEE, cycling XEE activate the SAC in response to unattached KTs. Purified p31^comet^ antagonizes Mad2 inhibition of APC/C^cdc20^ in XEE.^15^

We have used XEEs for investigation of the mitotic role(s) and regulation of p31^comet^. Here we show that p31^comet^ depletion from XEE caused a SAC-dependent delay in anaphase onset, suggesting that endogenous p31^comet^ is important for mitotic timing in this system. p31^comet^ was mitotically phosphorylated in XEE. While a number of well-established mitotic kinases did not efficiently modify p31^comet^ *in vitro*, IKK-β (Inhibitor of nuclear factor κ-B kinase-β) was effective for p31^comet^ phosphorylation. Depletion or inhibition of IKK-β delayed mitotic exit of XEEs, and a phosphomimetic p31^comet^ mutant showed increased activity in dissociation of soluble MCC and particularly in disruption of KT association of SAC components. Together, our results suggest that p31^comet^ contributes to the timing of anaphase onset in XEE through antagonism of the SAC and that IKK-β modifies p31^comet^ to enhance its activity.

## Results

### *Xenopus* p31^comet^ promotes SAC silencing in XEE

**Figure 1.**
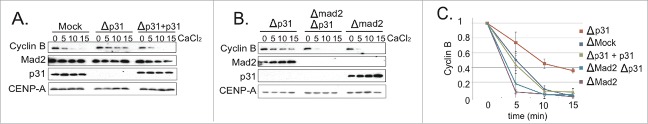
p31^comet^ depletion causes an SAC-dependent mitotic exit delay. (**A**) p31^comet^-depleted XEE reactions containing DSN, with or without 15 nM His_6_-p31^comet^ were incubated at 23°C for 15 min., followed by CaCl_2_ addition. At intervals after CaCl_2_ addition, samples were collected for Western blotting with the indicated antibodies. Mock lanes show samples from a mock-depleted reaction, without added His_6_-p31^comet^. (**B**) XEEs depleted of p31^comet^, Mad2 or both proteins were subjected to analysis as in (**A**). (**C**) Cyclin B levels were quantified for reactions as described in Panel A and B, and normalized relative to the initial level at time = 0. Values represent the mean ± SD derived from 3 independent assays.

To test whether p31^comet^ modulates mitotic exit timing in XEE under circumstances permissive for spindle assembly, we incubated control and p31^comet^-depleted CSF-XEE reactions containing DSN for 15 min. at 23°C, followed by CaCl_2_ addition to initiate mitotic exit. We examined the progression of each reaction into interphase through Western blotting for Cyclin B. Cyclin B degradation was significantly slower in p31^comet^-depleted samples (**Fig. 1A**) in a manner that could be rescued by addition of recombinant p31^comet^. These observations indicate that p31^comet^ facilitates mitotic exit in CSF-XEE containing DSN.

We postulated that transiently unattached KTs temporarily activate the SAC in XEEs, and that p31^comet^ promotes subsequent SAC silencing after MT-KT attachments were formed. To test this idea, we examined whether the delay in p31^comet^-depleted XEEs requires Mad2 (**Fig. 1B, C**). Mad2 depletion alone accelerated the rate of Cyclin B degradation, consistent with the notion that SAC activation modulates mitotic exit timing. The presence or absence of p31^comet^ did not significantly alter Cyclin B degradation in Mad2-depleted XEEs, indicating that p31^comet^ modulates mitotic exit through Mad2. Spindle morphology and MT density were indistinguishable in the presence and absence of p31^comet^ (**Fig. S1A**), arguing that p31^comet^ depletion did not delay mitotic exit by disrupting spindle assembly, and p31^comet^-depleted XEEs could activate their SAC in response to fully unattached KTs. (**Fig. S1B**).

Together, these observations suggest that the SAC slows mitotic exit in CSF-XEE containing DSN, and that p31^comet^ promotes anaphase by antagonizing the SAC. This observation is consistent with reports that p31^comet^-depleted HeLa cells show mitotic exit delays because they are unable to mediate SAC silencing.^8,11,16^

### p31^comet^ phosphorylation by IKK-β during mitotic exit

**Figure 2.**
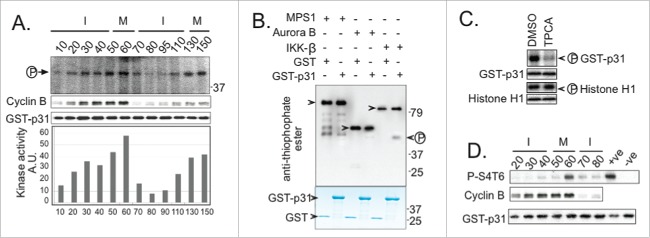
IKK-β phosphorylates *Xenopus* p31^comet^. (**A**) Samples from cycling XEEs with 1,000 DSN/µl were collected at intervals after shift to 23°C. GST-p31^comet^ beads and [γ−^32^P]ATP were added to an aliquot of the sample for analysis of kinase activity as described in Methods (top panel, bottom histogram). In parallel, another aliquot was directly analyzed by Western blotting for Cyclin B (second panel). The arrow indicates^32^P-labeled GST-p31^comet^. The stage of the cycling XEE is shown above (I: interphase, M: mitosis). (**B**) GST-p31^comet^ or GST were incubated with ATP-γ-S and Aurora B, Mps1 or IKK-β. Samples from each reaction were subject to Western blotting using an anti-thiophosphate ester specific antibody (upper panel) and to CBB staining (lower panel). Black arrows within the upper panel indicate bands resultant from kinase autophosphorylation. Arrow to the right of the upper panel indicates phosphorylated GST-p31^comet^. (**C**) CSF-XEE containing [γ−^32^P]ATP and either DMSO or TCPA (final concentration 300 µM) were incubated with GST-p31^comet^ or Histone H1. Each sample was subjected to SDS-PAGE and autoradiography (upper panels. Arrows indicate phosphorylated p31^comet^ or Histone H1) and to Western blotting to assure equal loading (lower panels). (**D**) Samples as in panel A were subjected to Western blotting using phospho-specific antibody (anti-p31^comet^-S4^p^,T6^p^). Positive control (+ve) lane contains *in vitro* phosphorylated GST-p31^comet^ as in **Figure S3A**, except ATP was used instead of [γ−^32^P]ATP. Negative control (-ve) lane includes untreated purified GST-p31^comet^.

It has been reported that mammalian p31^comet^ is phosphorylated during mitosis,^17,18^ and that phosphorylation of human p31^comet^ on Serine-102 weakens its interaction with Mad2 and its capacity to silence the SAC.^19^ We wished to determine whether p31^comet^ might be phosphorylated in XEE, and, if so, what role this modification might play in its activity. To address this question, we took samples from a cycling XEE reaction at different times, and examined the activity of kinases that can phosphorylate p31^comet^ by adding GST-p31^comet^ and [γ−^32^P]ATP to each sample. After 30 min., we re-isolated GST-p31^comet^ and subjected it to SDS-PAGE and autoradiography (**Fig. 2A**). We observed that kinase activity directed against GST-p31^comet^ increased progressively, peaking near the onset of Cyclin B degradation and mitotic exit in the intact cycling XEE (≈60 min.) and declining abruptly as the cycling XEE returned to interphase.

To determine which kinase mediates p31^comet^ phosphorylation, we examined the capacity of purified Mps1, Aurora B, Cyclin B/Cdk1 and IKK-β to phosphorylate GST-p31^comet^ (**Fig. 2B**, **Fig. S2**). Mps1, Aurora B and Cyclin B/Cdk1 are well-studied mitotic kinases. IKK-β has been implicated in spindle assembly^20^ and SAC regulation.^21^ The only kinase that efficiently phosphorylated GST-p31^comet^ *in vitro* was IKK-β. To test whether IKK-β has a major role in p31^comet^ phosphorylation, we performed kinase assays using CSF-XEE treated with IKK-β inhibitor TPCA ([5-(p-fluorophenyl)-2-ureido] thiophene-3-carboxamide).^22^ TPCA both inhibited phosphorylation of GST-p31^comet^ in CSF-XEE (**Fig. 2C**) and abolished IKK-β kinase activity toward GST-p31^comet^ reactions containing purified proteins (**Fig. S3A**). TPCA did not inhibit phosphorylation of the Cyclin B/Cdk1 substrate Histone H1 within CSF-XEE (**Fig. 2C**), arguing that it specifically blocked mitotic p31^comet^ phosphorylation through IKK-β inhibition.

We performed mass spectrometry to identify the sites on p31^comet^ phosphorylated by IKK-β *in vitro*, and identified Serine-4 (S4), Threonine-6 (T6) and Threonine-179 (T179) of p31^comet^ as candidate IKK-β sites (**Fig. S4A**). Mutation of either S4 and T6 to alanine or S179 to alanine reduced *in vitro* p31^comet^ phosphorylation, while mutation of all 3 residues to alanines nearly abolished p31^comet^ modification (**Fig. S4B**). We raised rabbit antibodies that specifically recognized p31^comet^ phosphorylated on S4 and T6 (p31^comet^-S4^p^,T6^p^): Anti-p31^comet^-S4^p^,T6^p^ antibodies did not recognize bacterially expressed GST-p31^comet^ on Western blots, but showed a prominent band of the appropriate molecular weight after GST-p31^comet^ was treated with IKK-β (**Fig. S3B**). The appearance of this band was suppressed by the addition of TPCA to the reaction. In a similar fashion, the anti-p31^comet^-S4^p^,T6^p^ antibodies recognized GST-p31^comet^ that had been incubated in XEE alone or in XEE plus DMSO, but not GST-p31^comet^ incubated in XEE reactions containing TPCA (**Fig. S3C**). We also raised rabbit antibodies against Xenopus IKK-β (anti-xIKK-β), which were used to immunodeplete IKK-β from XEE. We examined the capacity of these depleted XEE to phosphorylate GST-p31^comet^ by Western blotting with anti-p31^comet^-S4^p^,T6^p^ antibodies (**Fig. S3D**). We observed that phosphorylation of p31^comet^ was abolished after IKK-β depletion, but could be restored through the addition of purified human IKK-β at a similar concentration. These experiments collectively suggest that IKK-β phosphorylates p31^comet^ on S4 and T6, and that its kinase activity is essential for the modification of these residues in CSF-XEE.

In a manner similar to the experiment in **Fig. 2A**, we analyzed kinase activity against S4 and T6 of p31^comet^ in cycling XEE by Western blotting with anti-p31^comet^-S4^p^,T6^p^ antibodies (**Fig. 2D**). We observed that kinase activity against these residues peaked in mitosis, near the onset of Cyclin B degradation. TPCA abolished this peak of kinase activity toward p31^comet^-S4^p^,T6^p^ (**Fig. S3E**). Interestingly, the peak of anti-p31^comet^-S4^p^,T6^p^ kinase activity was somewhat sharper than overall kinase activity against p31^comet^ (compare **Fig. 2A and D**), potentially suggesting that p31^comet^ may be phosphorylated by other kinases during mitosis in addition to IKK-β. Our observations collectively indicate that p31^comet^ becomes phosphorylated near the onset of anaphase in XEE. Our data implicate IKK-β as a major kinase for at least a subset of these modifications, particularly S4 and T6.

### Phosphomimetic p31^comet^ antagonizes the SAC

**Figure 3.**
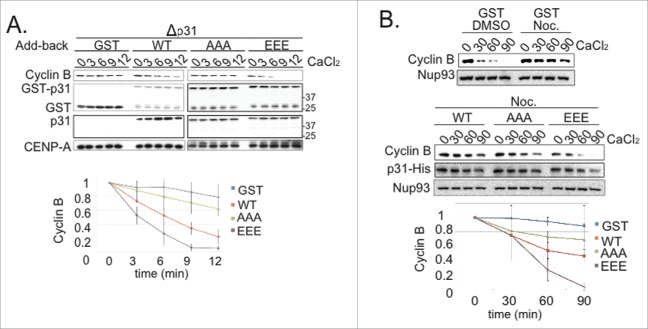
Phosphomimetic p31^comet^ accelerates M-phase exit. (**A**) 15 nM GST-tagged p31^comet-wt^, p31^comet-AAA^ or p31^comet-EEE^ were added to p31^comet^-depleted CSF-XEE containing DSN. The reactions were incubated for 20 min. at 23°C, followed by CaCl_2_ addition. Samples were taken at the indicated times after CaCl_2_ addition (in minutes), and subjected to Western blotting with anti-Cyclin B (top panel), anti-GST (second panel), anti-p31^comet^ (third panel) or anti-CENP-A (bottom panel, loading control). Graph shows Cyclin B levels, which were quantified at each point, and normalized relative to initial levels at time = 0. Values represent the mean ± SD derived from 3 independent assays. (**B**) 75 nM His-tagged p31^comet-wt^, p31^comet-AAA^ or p31^comet-EEE^ were added to p31^comet^-depleted CSF-XEE containing DSN, and, where indicated, 60 µM nocodazole (Noc.). The reactions were incubated for 30 min. at 23°C, followed by CaCl_2_ addition. Cyclin B destruction was monitored as in Panel A.

To examine whether p31^comet^ phosphorylation changes its activity, we depleted endogenous p31^comet^ from CSF-XEE, followed by addition of physiological levels (15 nM) of recombinant wild type p31^comet^ (p31^comet-wt^), a phosphomimetic form of p31^comet^, in which S4, T6 and T179 were mutated to glutamic acid (p31^comet-EEE^), or a non-phosphoryated form, in which those residues were mutated to alanine (p31^comet-AAA^). We added DSN to each reaction and incubated them at 23°C. After 15 min., we added CaCl_2_ and took periodic samples for Western blotting analysis of Cyclin B levels (**Fig. 3A**). p31^comet^ depletion delayed mitotic exit in a manner that was reversed by GST-p31^comet-wt^, but not by GST. GST-p31^comet-AAA^ was less effective than GST-p31^comet-wt^ at relieving this mitotic delay, while GST-p31^comet-EEE^ was more effective in accelerating Cyclin B degradation. Collectively, these findings are consistent with the idea that p31^comet^ phosphorylation enhances its capacity to promote mitotic exit.

To test the role of p31^comet^ phosphorylation in controlling its antagonism of the SAC *per se*, 75 nM p31^comet-wt^, p31^comet-AAA^ and p31^comet-EEE^ were added to p31^comet^-depleted CSF-XEE that also contained DSN and nocodazole. The reactions were incubated for 30 min. to allow SAC activation. CaCl_2_ was then added to cause CSF degradation, and mitotic exit was monitored by Western blotting with antibodies against Cyclin B (**Fig. 3B**). As expected, a control sample to which DMSO had been added in place of nocodazole exited from mitosis within 30 min. of CaCl_2_ addition. Cyclin B remained stable in a nocodazole-treated sample to which GST had been added, indicating the establishment of SAC arrest. The addition of p31^comet-AAA^ or p31^comet-wt^ weakly accelerated Cyclin B degradation in comparison to the nocodazole-treated control, while p31^comet-EEE^ promoted a faster rate of Cyclin B degradation. Our results are consistent with previous reports that excess p31^comet^ can block Mad2 function in XEEs,^15^ and indicate that p31^comet^ phosphorylation increases its effectiveness in disruption of the SAC.

### p31^comet^ phosphorylation alters Mad2 binding and KT interactions

**Figure 4.**
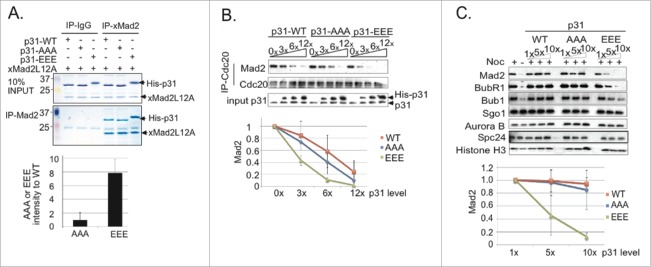
p31^comet^ phosphorylation by IKK**-β enhances** Mad2 association. (**A**) 50 nM recombinant His-tagged p31^comet-wt^, p31^comet-AAA^ or p31^comet-EEE^ were incubated with 100 nM recombinant *Xenopus* Mad2^L12A^. Mad2^L12A^ was immunoprecipitated using anti-Mad2 antibodies coupled to protein A Agarose beads. Mad2^L12A^ and co-precipitating p31^comet^ were detected by CBB staining. The level of each p31^comet^ variant was quantified and normalized to the amount of Mad2^L12A^. The relative yield of p31^comet-AAA^ or p31^comet-EEE^ was compared to the yield of p31^comet-wt^; the ratio of each mutant to the wild type protein is shown (lower graph). Values represent the mean ± SD from three independent assays. (**B**) His-tagged p31^comet-wt^, p31^comet-AAA^ or p31^comet-EEE^ were added at increasing concentrations (3× = 45 nM, 6× = 90 nM, 12× = 180 nM) to XEEs containing DSN and nocodazole. After 30 min. at 23°C, chromatin was removed by pelleting, and MCC was isolated from the soluble fraction by immunoprecipitation using anti-Cdc20 antibodies. Co-precipitating Mad2 was assayed by Western blotting. After quantification, Mad2 signals at each concentration for every p31^comet^ variant were normalized to the samples without p31^comet^ addition, as shown in the graph below. Values represent the mean ± SD from three independent assays. (**C**) Reactions were assembled as in (**B**), with increasing concentrations of His-tagged p31^comet-wt^, p31^comet-AAA^ or p31^comet-EEE^ (1× = 15 nM, 5× = 75 nM, 10× = 150 nM). After 30 m in. at 23°C, chromosomes were removed by pelleting and washed. The chromatin fraction was subjected to SDS-PAGE and Western blotting with the indicated antibodies. After quantification, Mad2 signals at each concentration for every p31^comet^ variant were normalized to the samples with 1× p31^comet^ (lower graph). Values represent the mean ± SD from three independent assays.

The ability of human p31^comet^ to disrupt the MCC requires c-Mad2 binding.^8,10,11^ To examine how p31^comet^ phosphorylation alters Mad2 binding, we incubated recombinant *Xenopus* Mad2^L12A^ with recombinant p31^comet-wt^, p31^comet-EEE^ or p31^comet-AAA^. Mad2^L12A^ is a Mad2 mutant that mimics c-Mad2.^10^ Mad2^L12A^ was immunoprecipitated from each reaction, and the bead-associated fractions were subjected to SDS-PAGE and Coomassie brilliant blue (CBB) staining (**Fig. 4A**). While the levels of p31^comet-wt^ and p31^comet-AAA^ precipitated were similar, p31^comet-EEE^ was more abundant, suggesting that it bound to Mad2^L12A^ more tightly.

We compared the capacity of recombinant p31^comet-wt^, p31^comet-AAA^ and p31^comet-EEE^ to release Mad2 from Cdc20 when added to CSF-XEE containing DSN and nocodazole. In each case, p31^comet^ was added at increasing concentrations and incubated for 30 min. at 23°C. Cdc20 was then immunoprecipitated from each sample, co-precipitating Mad2 was detected by immunoblotting (**Fig. 4B**). While all 3 forms of p31^comet^ disrupted the association of Cdc20 with Mad2, p31^comet-EEE^ was noticeably more effective. When we examined the capacity of p31^comet^ variants to release Mad2 from isolated MCC complexes (**Fig. S5A**), p31^comet-EEE^ was similarly more efficient than p31^comet-wt^ or p31^comet-AAA^. Together, these results suggest that p31^comet^ phosphorylation by IKK-β enhances its capacity for MCC dissociation, thus improving its capacity for SAC silencing.

To determine if IKK-β phosphorylation controls p31^comet^ activity at unattached KTs, we isolated chromatin from XEE incubated with nocodazole and p31^comet-wt^, p31^comet-AAA^ and p31^comet-EEE^. We analyzed the levels of KT-bound SAC components (Mad2, BubR1, Bub1), KT structural components (Spc24) and chromosome-associated inner centromeric proteins (Shugoshin1 and Aurora B).^23^ p31^comet-wt^ and p31^comet-AAA^ had little effect on the chromosome-bound levels of these components (**Fig. 4C**, **Fig. S5B**). By contrast, chromosomes showed a progressive loss of MCC components (Mad2, BubR1) and non-MCC SAC proteins (Bub1) with increasing p31^comet-EEE^ concentrations (**Fig. 4C**, **Fig. S5B**). p31^comet-EEE^ was not effective in releasing SAC components from KT in Mad2-depleted XEEs (**Fig. S5C**), consistent with the idea that p31^comet-EEE^ must associate with KT^8,11^ in order to act in this context. These data suggest Mad2-dependent p31^comet^ recruitment to KT allows it to play a positive role in dispersion of SAC proteins from KT, and that this function is enhanced by IKK-β phosphorylation. We did not observe appreciably higher levels of SAC proteins on KTs in p31^comet^-depleted XEEs than in control samples, so we do not believe that p31^comet^ normally determines the maximal amount of their recruitment.

### Depletion or inhibition of IKK-β compromises SAC silencing

**Figure 5.**
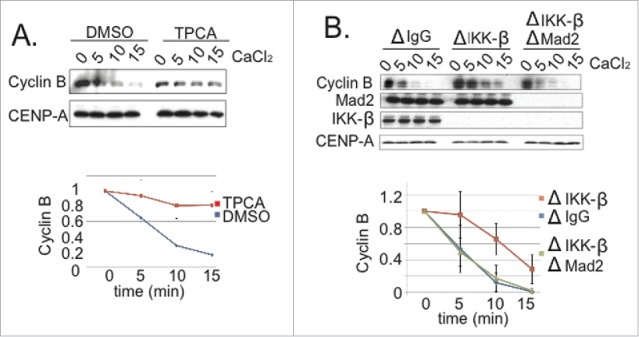
IKK-**β** loss compromises SAC silencing. (**A**) DMSO or TPCA (final concentration 300 µM) were added to CSF-XEE with DSN. The reactions were incubated for 10 min. at 23°C before CaCl_2_ addition. Samples were taken at indicated times after CaCl_2_ addition, and subjected to SDS-PAGE and Western blotting with anti-Cyclin B and -CENP-A (loading control) antibodies. The graph shows Cyclin B levels, which were quantified in each reaction and normalized relative to the initial level at time = 0. Values represent the mean ± SD derived from 3 independent assays. (**B**) XEEs were depleted of IKK-β, or both IKK-β and Mad2. Reactions were carried out as in Panel A, and samples were subjected to SDS-PAGE and Western blotting with the indicated antibodies. The graph shows Cyclin B levels, which were quantified in each reaction and normalized relative to the initial level at time = 0. Values represent the mean ± SD derived from 3 independent assays. ΔIgG lanes show samples from a mock-depleted reaction.

Our findings indicated that p31^comet^ plays an important role in anaphase onset in XEE, and that its activity is significantly enhanced through phosphorylation by IKK-β. Together, these observations predicted that IKK-β should promote anaphase in XEE. To directly test this prediction, we examined the consequences of IKK-β inhibition or depletion upon mitotic exit in CSF-XEE. First, we added TPCA to CSF-XEE containing DSN. After 20 min. at 23°C, we added CaCl_2_ and followed mitotic exit (**Fig. 5A**). We observed slower Cyclin B degradation in the TPCA-treated reaction, indicating that IKK-β inhibition delays mitotic exit. Second, we examined mitotic exit in IKK-β-depleted CSF-XEE containing DSN. Spindles appeared normal in the absence of IKK-β before CaCl_2_ addition (**Fig. S6B**). However, mitotic exit was significantly delayed after CaCl_2_ addition, with longer persistence of both high Cyclin B levels (**Fig. 5B**) and spindle MTs (**Fig. S6B**). This delay was reversed through the addition of purified human IKK-β (**Fig. S6A**). As with the delay caused by p31^comet^ depletion (**Figure 1B, C**), co-depletion of Mad2 restored anaphase progression in IKK-β-depleted XEEs (**Fig. 5B**), consistent with the idea that IKK-β accelerates mitotic exit by antagonizing the SAC through p31^comet^ phosphorylation.

## Discussion

SAC activation has been shown to occur transiently during each cell cycle in other metazoan systems,^5,6^ even in the absence of external perturbations, so that subsequent SAC silencing is pivotal in determining the timing of anaphase. Our data indicate that similar regulation can be recapitulated within the XEE *in vitro* system (**Fig. 1**): Under the conditions that we have used, Mad2 depletion accelerated Cyclin B degradation in XEE, indicating that the SAC acts as a brake to anaphase progression. Moreover, p31^comet^, a protein required for SAC silencing, promoted anaphase in this system. Since the presence or absence of p31^comet^ did not modulate anaphase timing in the absence of Mad2, our data suggest that p31^comet^ acts primarily by antagonizing the SAC. Furthermore, we found that kinase activity directed against p31^comet^ increased near anaphase onset in XEE, and that IKK-β was among the principal kinases that modify p31^comet^ in this interval (**Fig. 2**). Analysis of phosphomimetic p31^comet^ mutants showed that p31^comet^ phosphorylation promotes its activity in SAC silencing (**Figs. 3, 4**), while the depletion or inhibition of IKK-β reduced p31^comet^'s capacity to act in this context (**Fig. 5**).

p31^comet^ has been reported to act both on soluble MCC^11^ and at KTs,^10,13^ and we find evidence consistent with both modes of action in XEE (**Fig. 4**, **Fig. S5**). Notably, while p31^comet-EEE^ was somewhat more efficient than p31^comet-wt^ at mediating the dissociation of Mad2 from Cdc20 within the soluble faction (**Fig. 4B**), its release of SAC proteins from unattached KTs was dramatically better than p31^comet-wt^ (**Fig. 4C**), suggesting that p31^comet^ phosphorylation by IKK-β particularly enhances its capacity to act at KT. We estimate that the concentration of p31^comet^ (15 nM) is much lower than the concentration of Mad2 (300 nM) within XEE (**Fig. S7**). Because of this difference, endogenous p31^comet^ may only be sufficient to neutralize a small fraction of the soluble Mad2 through stoichiometric binding.^13^ It is interesting to speculate that p31^comet^ phosphorylation may promote its activity specifically against the smaller pool of Mad2 associated to KT, and that this might be its primary site of action within XEE.

Our findings are interesting in light of the underappreciated role of IKK-β within mitosis, which is distinct from its function in cellular stress and inflammatory signaling pathways.^24^ IKK-β depletion causes mitotic delays in mammalian cells, accompanied by spindle assembly defects and stabilization of the Aurora A kinase.^20^ On the other hand, the IKK-β inhibitor BMS-345541 causes precocious APC/C activation in tissue culture cells, resulting in early mitotic exit, defective chromosome separation and improper cytokinesis, and BMS-345541 can override the SAC in nocodazole-arrested cells.^21^ It is notable in this context that the transcription factor NF-κB, a critical IKK-β target, controls the expression of numerous cell cycle regulators.^24^ NF-κB's activity is stimulated by drugs that act on the MT cytoskeleton, including taxol or nocodazole,^25,26^ suggesting the interesting possibility that the SAC may regulate IKK-β.

It is not clear why IKK-β depletion^20^ and its chemical inhibition^21^ have opposite effects on mitotic exit in mammalian cells, with the former causing mitotic arrest while the latter promotes SAC release. These distinct phenotypes may reflect the activity of IKK-β against multiple downstream targets. Within XEEs, a simpler system that does not require gene expression,^14^ IKK-β depletion and its inhibition by TPCA both caused mitotic exit delays (**Fig. 5**). It seems likely that p31^comet^ phosphorylation by IKK-β contributes to its capacity to promote mitotic exit in XEE, since phosphomimetic p31^comet-EEE^ was more effective than wild type p31^comet^ in a variety of assays for Mad2 binding and SAC release (**Figs. 3, 4**). Since structural analysis of the complex between Mad2 with p31^comet^ did not resolve the first 53 residues within p31^comet^'s N-terminal domain,^10^ it is difficult to speculate mechanistically as to how phosphorylation of this region might enhance the stability of Mad2-p31^comet^ association. The N-terminus of Xenopus p31^comet^ is not closely conserved with mammalian p31^comet^ homologues (**Fig. S8**). Nevertheless, alignment of their sequences shows that mouse p31^comet^ has residues that may correspond to the S4 and T6 phosphorylation sites, while human p31^comet^ shares the site corresponding to T6. This relationship suggests that p31^comet^ phosphorylation by IKK-β could be important for mitotic control in mammals as well as amphibians, a possibility that we are currently testing.

In summary, our results show that the SAC can modulate anaphase timing in XEE without external perturbation of spindle assembly, and suggest a model in which IKK-β enhances mitotic exit in XEE at least partially through phosphorylation and activation of p31^comet^ as a SAC release factor. While previous reports have indicated a role of IKK-β within mitosis, our findings are among the first mechanistic insights into the nature of this role. These observations point toward an interesting possible convergence of two disparate fields through the action of a classical signaling kinase upon the mitotic machinery.

## Materials and Methods

### Constructs and protein purification

We performed a blast search using the human p31^comet^ protein sequence against the *X. laevis* and X. *tropicalis* mRNA plus EST cluster database (protein query to DNA database) to identify the Xenopus homolog of p31^comet^. The cDNA sequence that we retrieved (Genbank: CA973841.1) encodes a protein that is 52% identical and 64% similar to the human p31^comet^ protein (**Fig. S8**).

A cDNA encoding wild type *Xenopus* p31^comet^ was amplified from a *X*. laevis cDNA library using forward (ATGGCGCAGAGTGGCACAGATCTACC) and reverse (TCAGTTGTGAAACCCCTTTACTAT) primers. It was subcloned into a pGEX4T1 vector for protein expression in *E. Coli*, or into pFastBac HT for expression in Baculovirus infected insect cells. p31^comet^ phosphomimetic and phosphodeficient mutants were generated using PCR-based site-directed mutagenesis. *Xenopus* Mad2 was cloned into pGEX4T1 using forward (ATGTCGTCCATCTTGCCTTTCACCCCGCCAGTAG) and reverse (TTAGGACATGCTTGAGCAGCGGACTGAAGGGGATCCCATCTGTGT) primers. *Xenopus* Mad2L12A was generated by site direct mutagenesis.

GST-tagged p31^comet^ variants and Mad2 were purified from *E. Coli* using glutathione Sepharose beads (Amersham). The GST tag of GST-Mad2L12A was subsequently removed by thrombin digestion. His_6_-tagged p31^comet^ variants were purified from Baculovirus infected insect cell lysates using Ni^2+^-NTA resin (Qiagen).

### Antibodies, Western blotting and kinases

Rabbit anti-*Xenopus* p31^comet^, anti-*Xenopus* Mad2 and anti-*Xenopus* IKK-β were raised by Pacific Immunology Corporation (Ramona, CA) against p31^comet^ (full length), Mad2 (full length) and IKK-β (aa 100–300) proteins. Anti-peptide antibodies that specifically recognize p31^comet^ phosphorylated on residues S4 and T6 were generated in rabbits by the Pacific Immunology Corporation using peptide MAQ-Sp-G-Tp-DLPLRRAH.

Antibodies were used at the following concentrations: anti-p31^comet^ (1:1,000, rabbit), anti- IKKβ (1:1000, rabbit), anti-*Xenopus* cyclin B (1:1,000 rabbit, Abcam), anti-CENP-A (1:1,000 rabbit), anti-Mad1 (1:1,000 rabbit) and anti-GST (1:5,000 mouse, Santa Cruz), anti-xMad2 (1:1,000 rabbit), anti-BubR1 (1:1,000 rabbit for western blotting and 1:300 chicken for IF), anti-Cdc20 (1:1,000 rabbit), anti-Spc24 (1:1,000 rabbit), anti-Bub1 (1:1000 rabbit), anti-Aurora B (1:1,000 rabbit), Histone H3 (1:5,000 rabbit, SignalChem), anti-Shugoshin (1:1,000 rabbit), anti-Nup93 (1:1,000 rabbit).

For quantification of Western blots in **Figure 1**, 3,5 and 4A films were scanned and ImageJ software was used to quantify respective Western blot bands in the resultant images. For quantification of Western blots in **Figure 4C** and **Figure S5A**, images were captured with Gel Logic 6000 Pro system (Carestream) and quantified using Carestream Imaging software.

Kinases (Mps1, Aurora B, IKK-β, Cdk1-Cyclin B) were purchased from SignalChem.

### Kinase assays

In **Figure 2A**, 2 µl of XEEs samples were incubated with 1 µCu [γ−^32^P]ATP and 2.5 µg GST-p31^comet^ in a final volume of 25 µl of reaction buffer (40 mM Hepes pH 7.8, 100 mM NaCl, 2 mM MgCl_2_, 40 mM β–glycerolphosphate). Reactions were incubated for 1 hr at 23°C. GST-p31^comet^ was precipitated on glutathione sepharose beads, and washed 3 times with buffer A (50 mM Tris-Cl pH 7.5, 150 mM NaCl, 0.05% Tween-20, protease inhibitor (EGTA-free tablet, Roche), 0.1 µM Okadic acid, 40 mM β–glycerolphosphate) for 4 times (5 min each). The samples were subjected to SDS-PAGE and^32^P labeled GST-p31^comet^ was visualized using a phosphoimager and captured by phospho-scanner. GST-p31^comet^ panel shows the kinase reaction blotted with anti-GST antibodies (third panel).

In **Figure 2B**, reactions included 500 µM ATP-γ-S, 100 ng/µl GST-p31^comet^ and 4 ng/ul (of each of each purified kinase, as indicated, in a final volume of 25 µl of reaction buffer (10 mM Tris-Cl pH 7.5, 150 mM NaCl, 10 mM MgCl_2_). The reactions were incubated for 1 hour at 23°C, and terminated though addition of 20 mM EDTA. 2.5 mM p-nitrobenzyl mesylate (PNBM) was added to each sample, followed by an additional hour of incubation at 23°C. The samples were subjected to SDS-PAGE and Western blotting using the thiophosphate ester specific rabbit (Abcam) antibodies to identify phosphorylated proteins.

### Preparation and use of XEEs

CSF-XEEs, cycling XEEs and DSN were prepared as described.^27,14^ Unless otherwise indicated, DSN were added at 10,000 DSN/µl, and CaCl_2_ was added to a concentration of 0.6 mM to promote mitotic exit of CSF-XEEs. Where indicated, nocodazole was added at 65 µM. Immunodepletions of p31^comet^, IKKβ and Mad2 were made using antibodies coupled to protein-A Dynal magnetic beads (Life Technologies) through 2 round of depletion. The p31^comet^ variants were added to depleted XEEs when indicated. In **Figure 4C**, isolated chromatin was prepared and analyzed as previously described.^27^

To assay MCC disruption in **Figure 4B**, His_6_-tagged p31^comet-wt^, p31^comet-AAA^ or p31^comet-EEE^ were added at the indicated concentrations to CSF-XEE containing DSN and nocodazole, and incubated at 23°C for 30 min. MCC was immunoprecipitated from each sample using anti-Xenopus Cdc20 antibodies coupled to protein-A Dynal magnetic beads. The beads were washed 3 times with buffer A, eluted with SDS sample buffer and subjected to Western blotting.

### *In vitro* binding of recombinant Mad2 and p31^comet^ variants

In **Figure 4A**, 100 nM Mad2 and 50 nM His_6_-tagged p31^comet-wt^, p31^comet-AAA^ or p31^comet-EEE^ were incubated in 1 ml of TBS with 0.05% Tween20 (TBST) buffer at 23°C for 1 hour. Mad2 was Immunoprecipitated using anti-Mad2 antibodies coupled to Protein A-agarose beads. The beads were washed 3 times using Buffer A and the associated proteins were visualized by SDS-PAGE and CBB staining.
