# Supplementary material for: Phosphorylation of Xenopus p31comet potentiates mitotic checkpoint exit
Source: Cell Cycle. 2015 Apr 18;14(24):3978–85. doi: 10.1080/15384101.2015.1033590 (PMC4825729; doi:10.1080/15384101.2015.1033590)
Supplement: 1033590_supplemental_files.zip [file kccy-14-24-1033590-s001.zip › 1033590 supplemental files.pptx]

## Slide 1
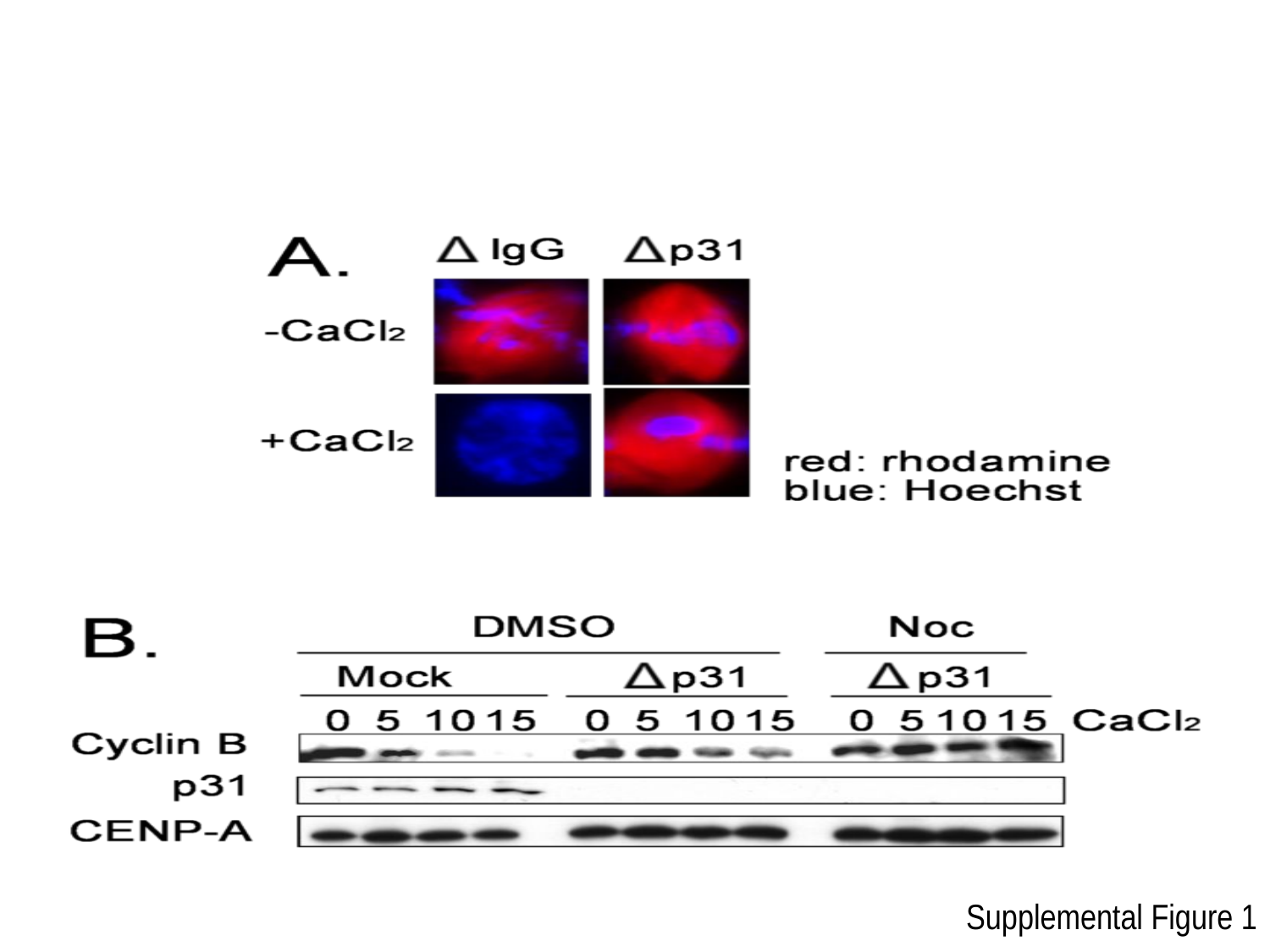

# Supplemental Figure 1

## Slide 2
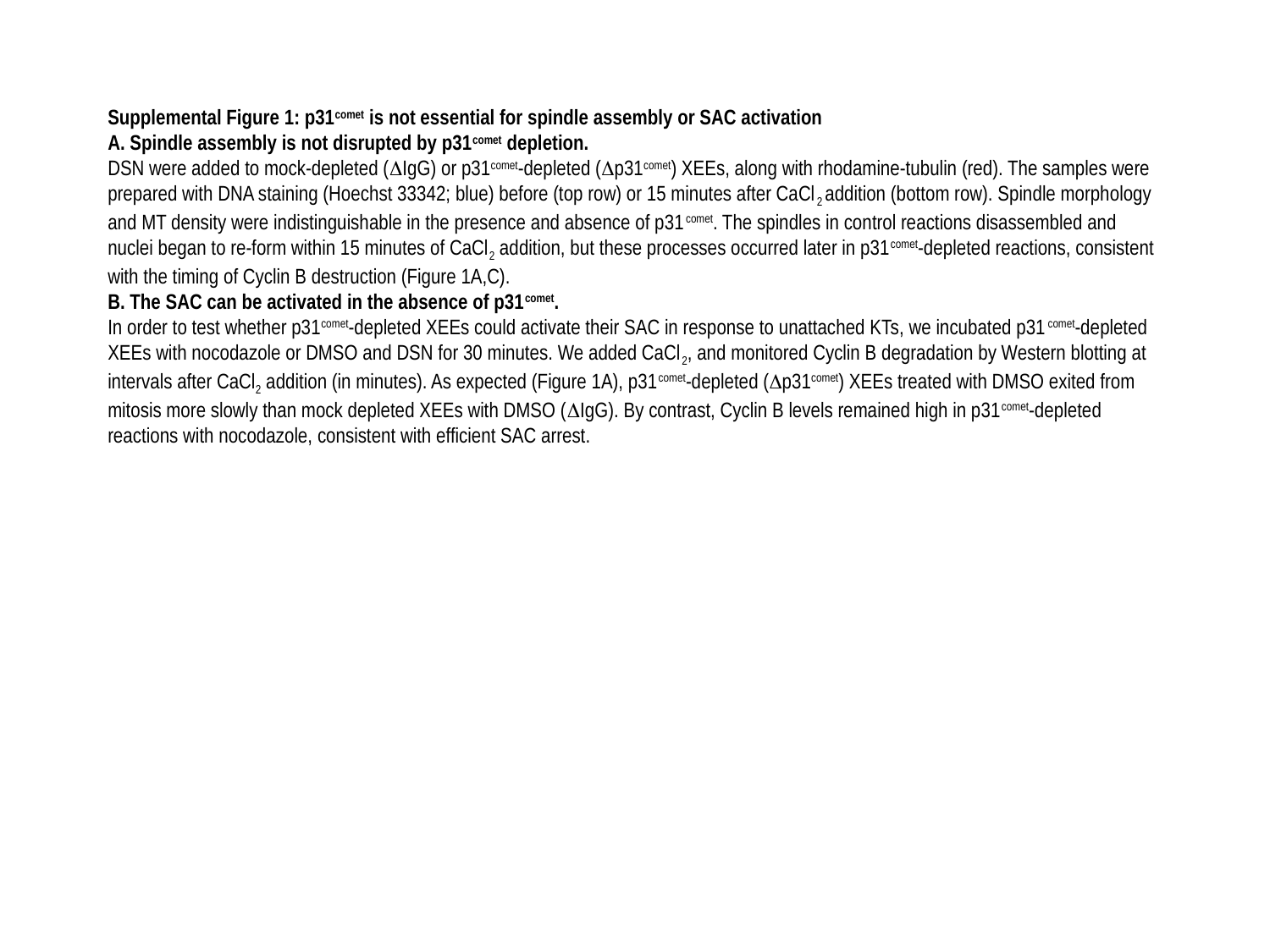

Supplemental Figure 1: p31comet is not essential for spindle assembly or SAC activation
A. Spindle assembly is not disrupted by p31comet depletion.
DSN were added to mock-depleted (ΔIgG) or p31comet-depleted (Δp31comet) XEEs, along with rhodamine-tubulin (red). The samples were prepared with DNA staining (Hoechst 33342; blue) before (top row) or 15 minutes after CaCl2 addition (bottom row). Spindle morphology and MT density were indistinguishable in the presence and absence of p31comet. The spindles in control reactions disassembled and nuclei began to re-form within 15 minutes of CaCl2 addition, but these processes occurred later in p31comet-depleted reactions, consistent with the timing of Cyclin B destruction (Figure 1A,C).
B. The SAC can be activated in the absence of p31comet.
In order to test whether p31comet-depleted XEEs could activate their SAC in response to unattached KTs, we incubated p31comet-depleted XEEs with nocodazole or DMSO and DSN for 30 minutes. We added CaCl2, and monitored Cyclin B degradation by Western blotting at intervals after CaCl2 addition (in minutes). As expected (Figure 1A), p31comet-depleted (Δp31comet) XEEs treated with DMSO exited from mitosis more slowly than mock depleted XEEs with DMSO (ΔIgG). By contrast, Cyclin B levels remained high in p31comet-depleted reactions with nocodazole, consistent with efficient SAC arrest.

## Slide 3
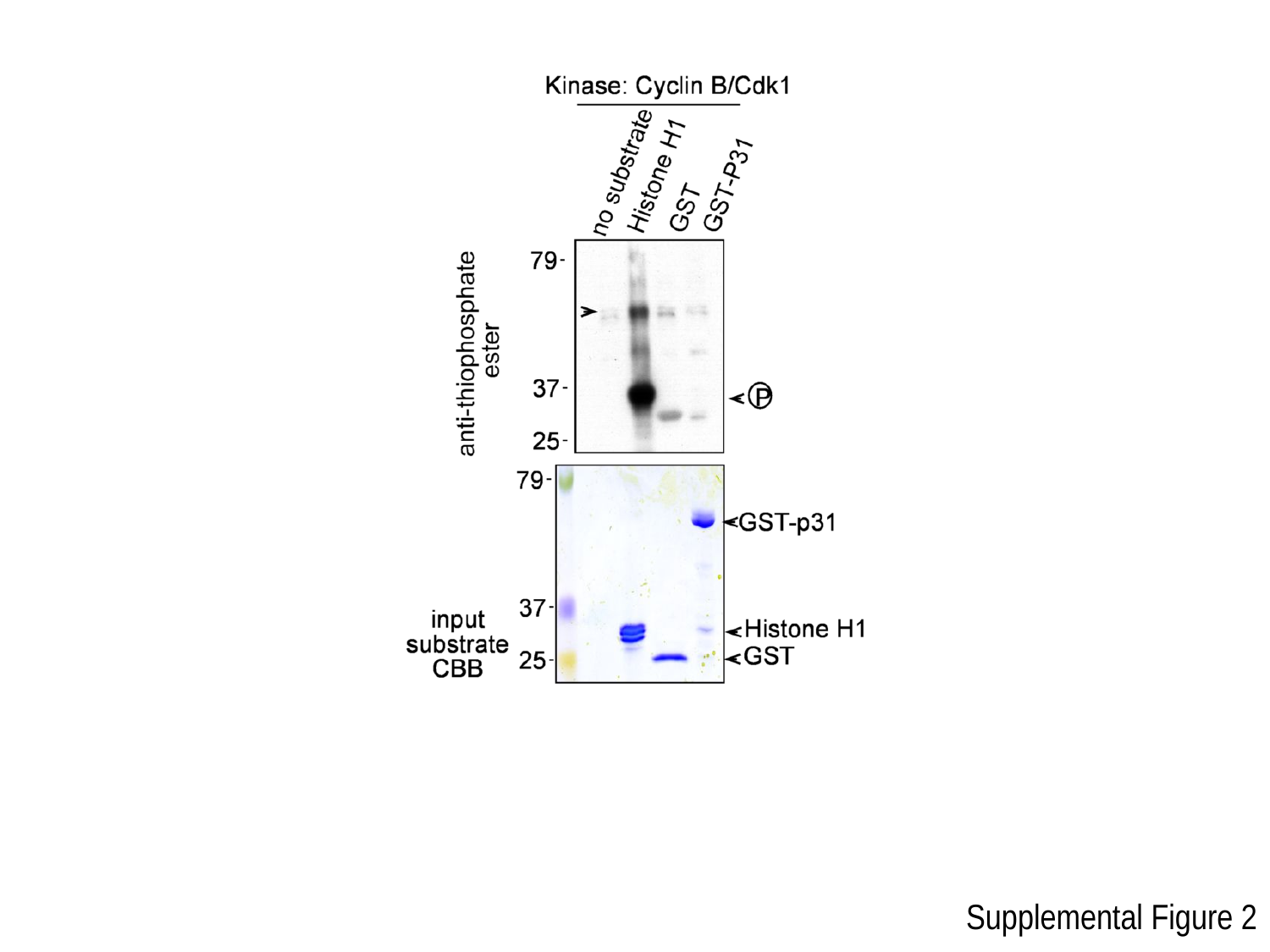

# Supplemental Figure 2

## Slide 4
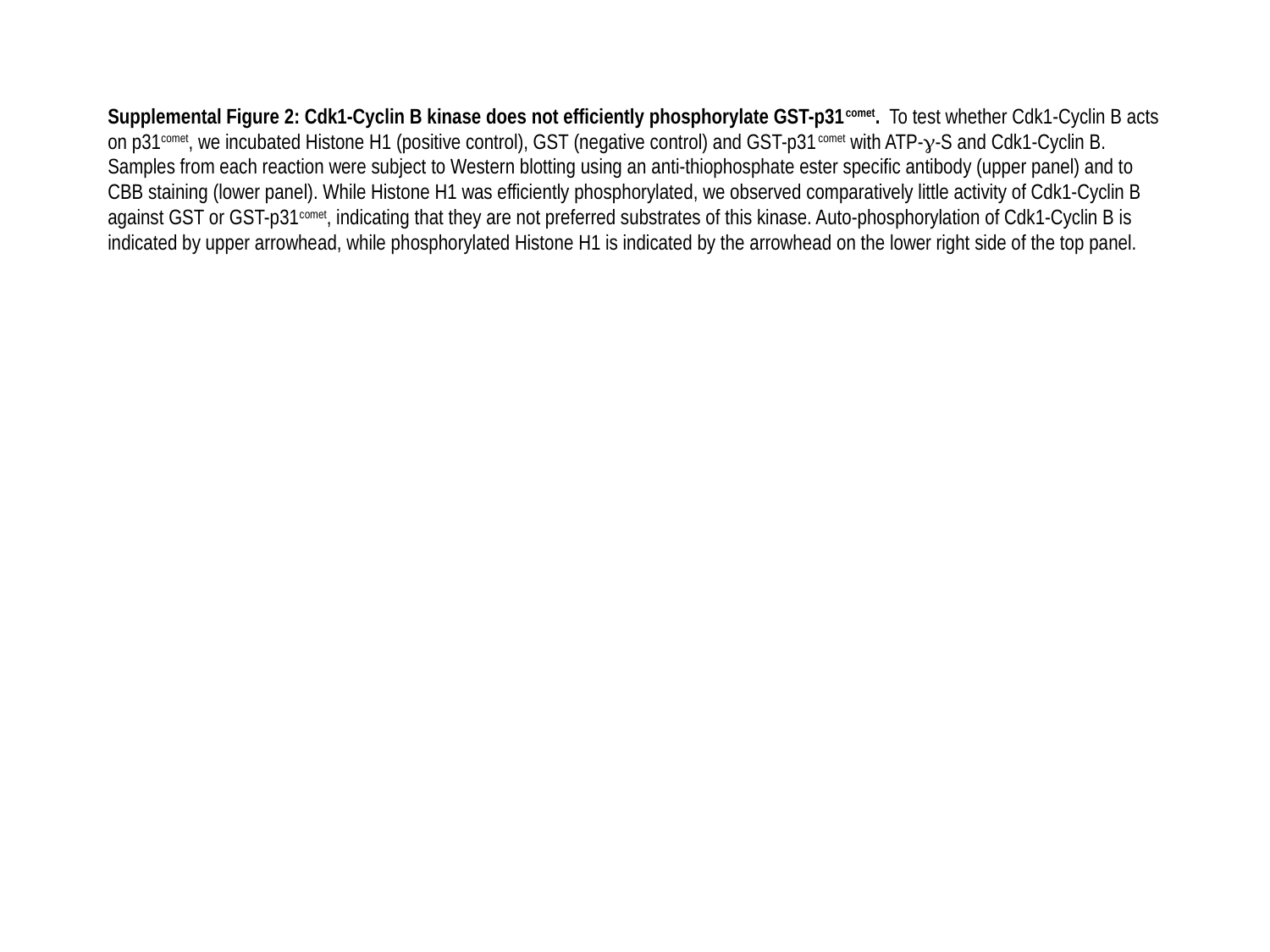

Supplemental Figure 2: Cdk1-Cyclin B kinase does not efficiently phosphorylate GST-p31comet. To test whether Cdk1-Cyclin B acts on p31comet, we incubated Histone H1 (positive control), GST (negative control) and GST-p31comet with ATP-γ-S and Cdk1-Cyclin B. Samples from each reaction were subject to Western blotting using an anti-thiophosphate ester specific antibody (upper panel) and to CBB staining (lower panel). While Histone H1 was efficiently phosphorylated, we observed comparatively little activity of Cdk1-Cyclin B against GST or GST-p31comet, indicating that they are not preferred substrates of this kinase. Auto-phosphorylation of Cdk1-Cyclin B is indicated by upper arrowhead, while phosphorylated Histone H1 is indicated by the arrowhead on the lower right side of the top panel.

## Slide 5
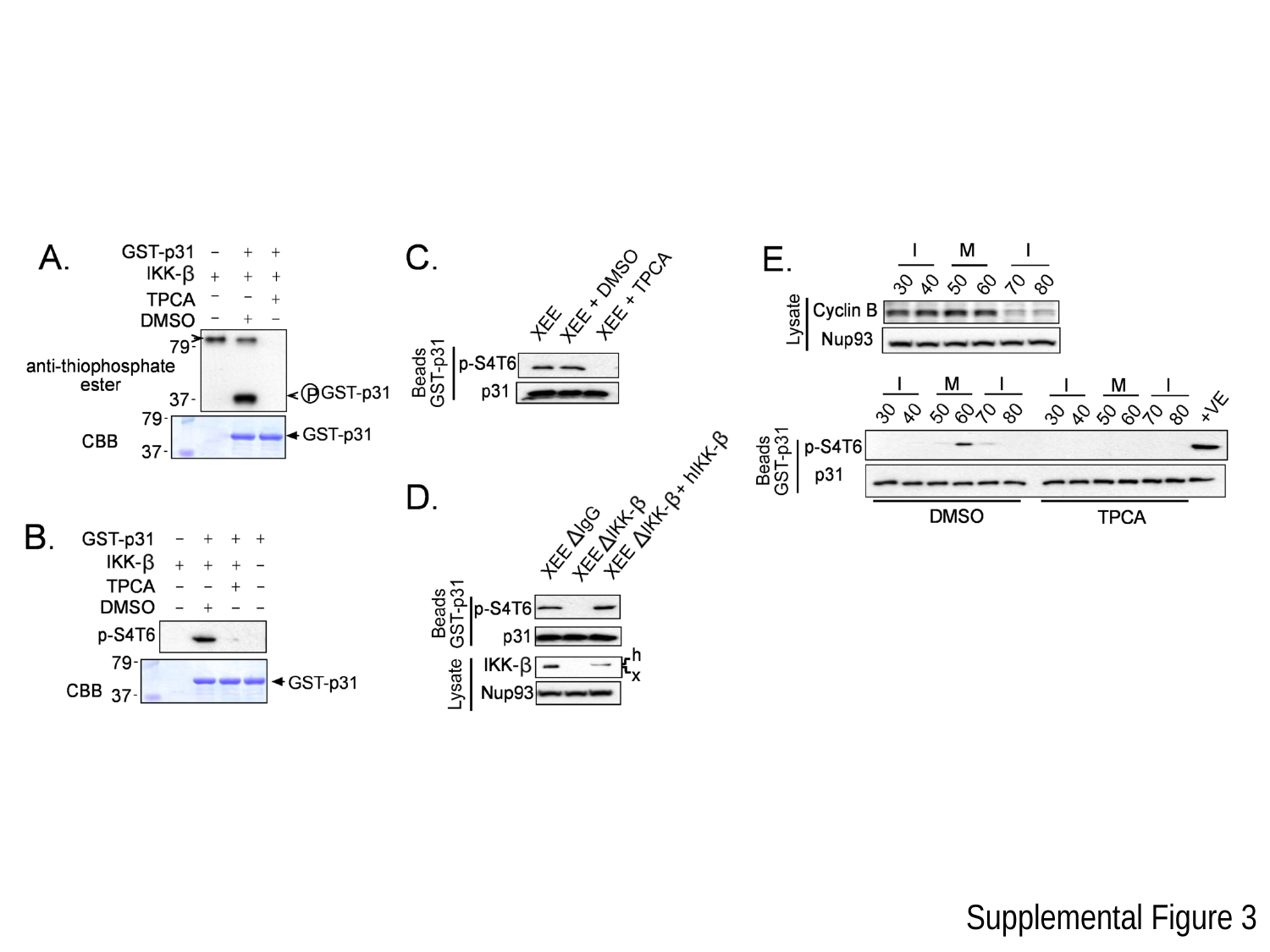

# Supplemental Figure 3

## Slide 6
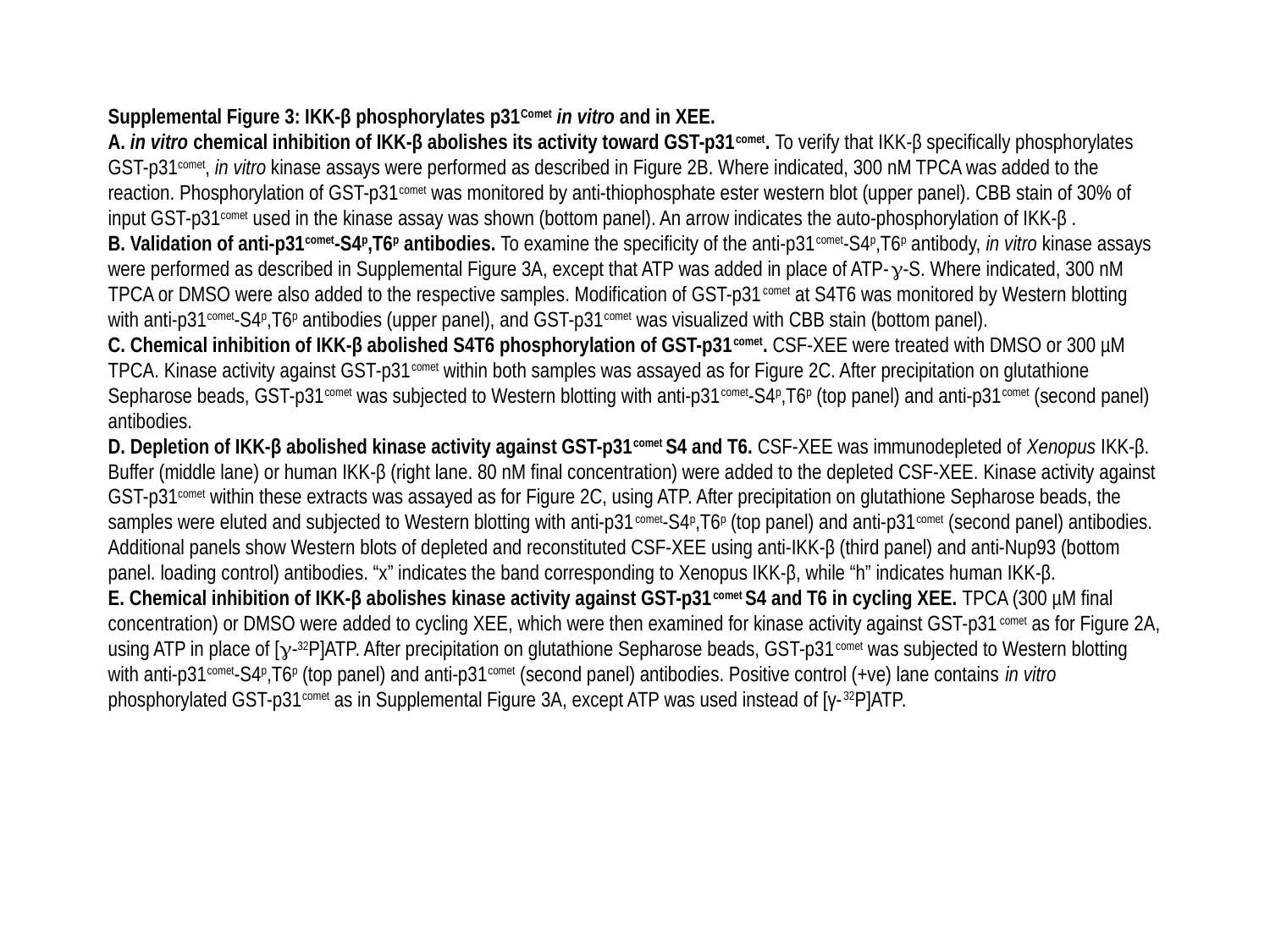

Supplemental Figure 3: IKK-β phosphorylates p31Comet in vitro and in XEE.
A. in vitro chemical inhibition of IKK-β abolishes its activity toward GST-p31comet. To verify that IKK-β specifically phosphorylates GST-p31comet, in vitro kinase assays were performed as described in Figure 2B. Where indicated, 300 nM TPCA was added to the reaction. Phosphorylation of GST-p31comet was monitored by anti-thiophosphate ester western blot (upper panel). CBB stain of 30% of input GST-p31comet used in the kinase assay was shown (bottom panel). An arrow indicates the auto-phosphorylation of IKK-β .
B. Validation of anti-p31comet-S4p,T6p antibodies. To examine the specificity of the anti-p31comet-S4p,T6p antibody, in vitro kinase assays were performed as described in Supplemental Figure 3A, except that ATP was added in place of ATP-γ-S. Where indicated, 300 nM TPCA or DMSO were also added to the respective samples. Modification of GST-p31comet at S4T6 was monitored by Western blotting with anti-p31comet-S4p,T6p antibodies (upper panel), and GST-p31comet was visualized with CBB stain (bottom panel).
C. Chemical inhibition of IKK-β abolished S4T6 phosphorylation of GST-p31comet. CSF-XEE were treated with DMSO or 300 µM TPCA. Kinase activity against GST-p31comet within both samples was assayed as for Figure 2C. After precipitation on glutathione Sepharose beads, GST-p31comet was subjected to Western blotting with anti-p31comet-S4p,T6p (top panel) and anti-p31comet (second panel) antibodies.
D. Depletion of IKK-β abolished kinase activity against GST-p31comet S4 and T6. CSF-XEE was immunodepleted of Xenopus IKK-β. Buffer (middle lane) or human IKK-β (right lane. 80 nM final concentration) were added to the depleted CSF-XEE. Kinase activity against GST-p31comet within these extracts was assayed as for Figure 2C, using ATP. After precipitation on glutathione Sepharose beads, the samples were eluted and subjected to Western blotting with anti-p31comet-S4p,T6p (top panel) and anti-p31comet (second panel) antibodies. Additional panels show Western blots of depleted and reconstituted CSF-XEE using anti-IKK-β (third panel) and anti-Nup93 (bottom panel. loading control) antibodies. “x” indicates the band corresponding to Xenopus IKK-β, while “h” indicates human IKK-β.
E. Chemical inhibition of IKK-β abolishes kinase activity against GST-p31comet S4 and T6 in cycling XEE. TPCA (300 µM final concentration) or DMSO were added to cycling XEE, which were then examined for kinase activity against GST-p31comet as for Figure 2A, using ATP in place of [γ-32P]ATP. After precipitation on glutathione Sepharose beads, GST-p31comet was subjected to Western blotting with anti-p31comet-S4p,T6p (top panel) and anti-p31comet (second panel) antibodies. Positive control (+ve) lane contains in vitro phosphorylated GST-p31comet as in Supplemental Figure 3A, except ATP was used instead of [γ-32P]ATP.

## Slide 7
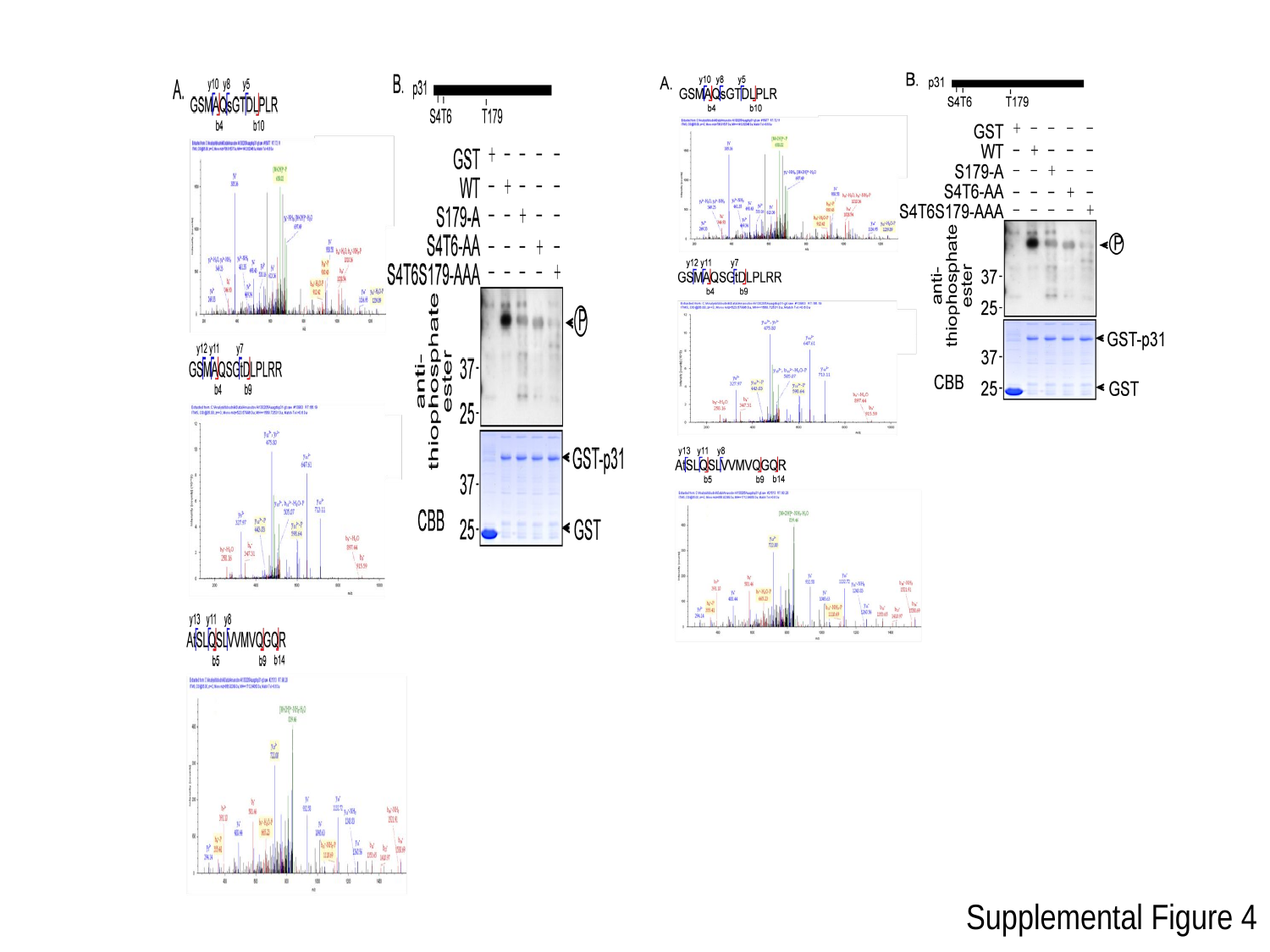

# Supplemental Figure 4

## Slide 8
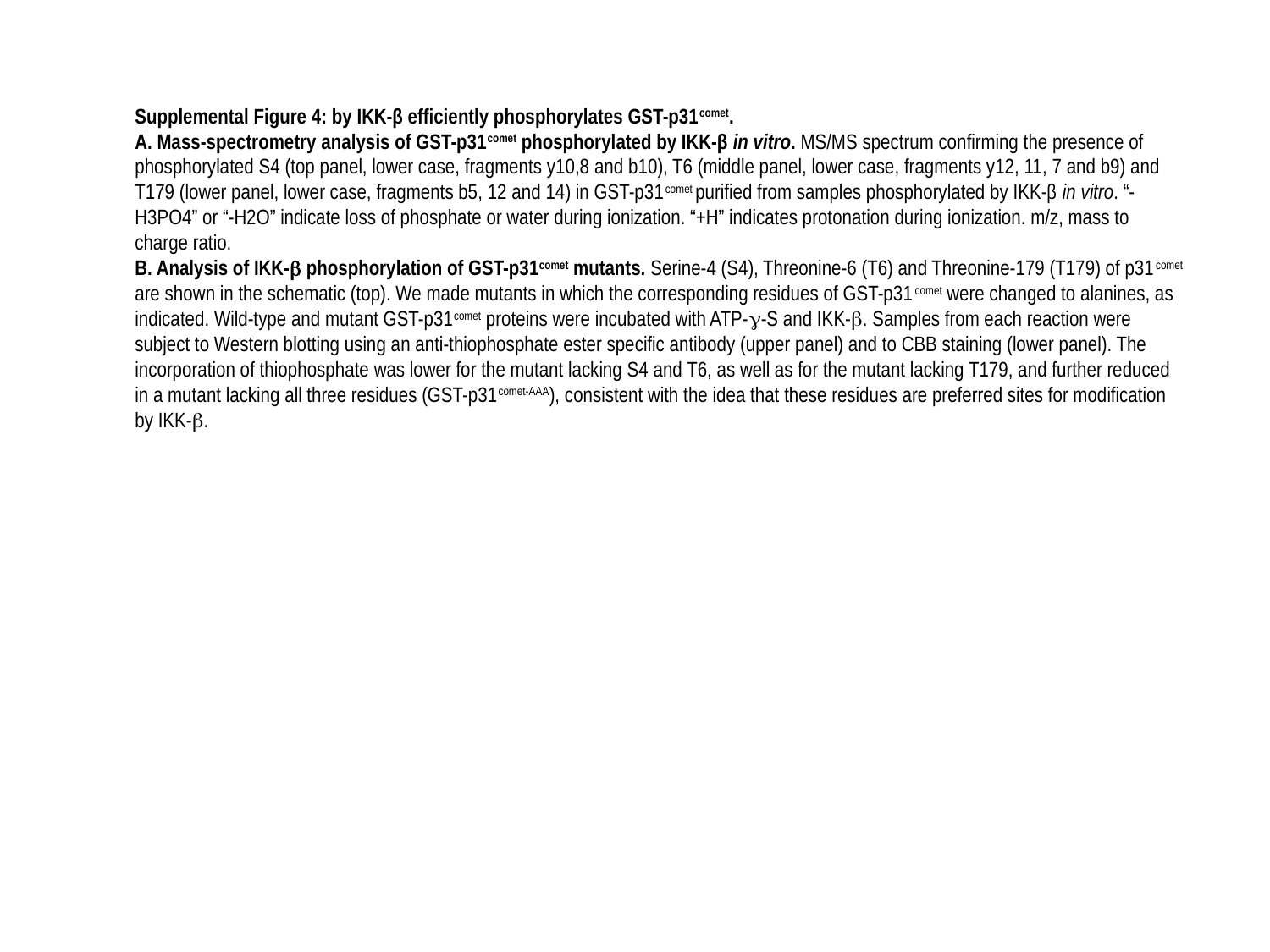

Supplemental Figure 4: by IKK-β efficiently phosphorylates GST-p31comet.
A. Mass-spectrometry analysis of GST-p31comet phosphorylated by IKK-β in vitro. MS/MS spectrum confirming the presence of phosphorylated S4 (top panel, lower case, fragments y10,8 and b10), T6 (middle panel, lower case, fragments y12, 11, 7 and b9) and T179 (lower panel, lower case, fragments b5, 12 and 14) in GST-p31comet purified from samples phosphorylated by IKK-β in vitro. “-H3PO4” or “-H2O” indicate loss of phosphate or water during ionization. “+H” indicates protonation during ionization. m/z, mass to charge ratio.
B. Analysis of IKK-β phosphorylation of GST-p31comet mutants. Serine-4 (S4), Threonine-6 (T6) and Threonine-179 (T179) of p31comet are shown in the schematic (top). We made mutants in which the corresponding residues of GST-p31comet were changed to alanines, as indicated. Wild-type and mutant GST-p31comet proteins were incubated with ATP-γ-S and IKK-β. Samples from each reaction were subject to Western blotting using an anti-thiophosphate ester specific antibody (upper panel) and to CBB staining (lower panel). The incorporation of thiophosphate was lower for the mutant lacking S4 and T6, as well as for the mutant lacking T179, and further reduced in a mutant lacking all three residues (GST-p31comet-AAA), consistent with the idea that these residues are preferred sites for modification by IKK-β.

## Slide 9
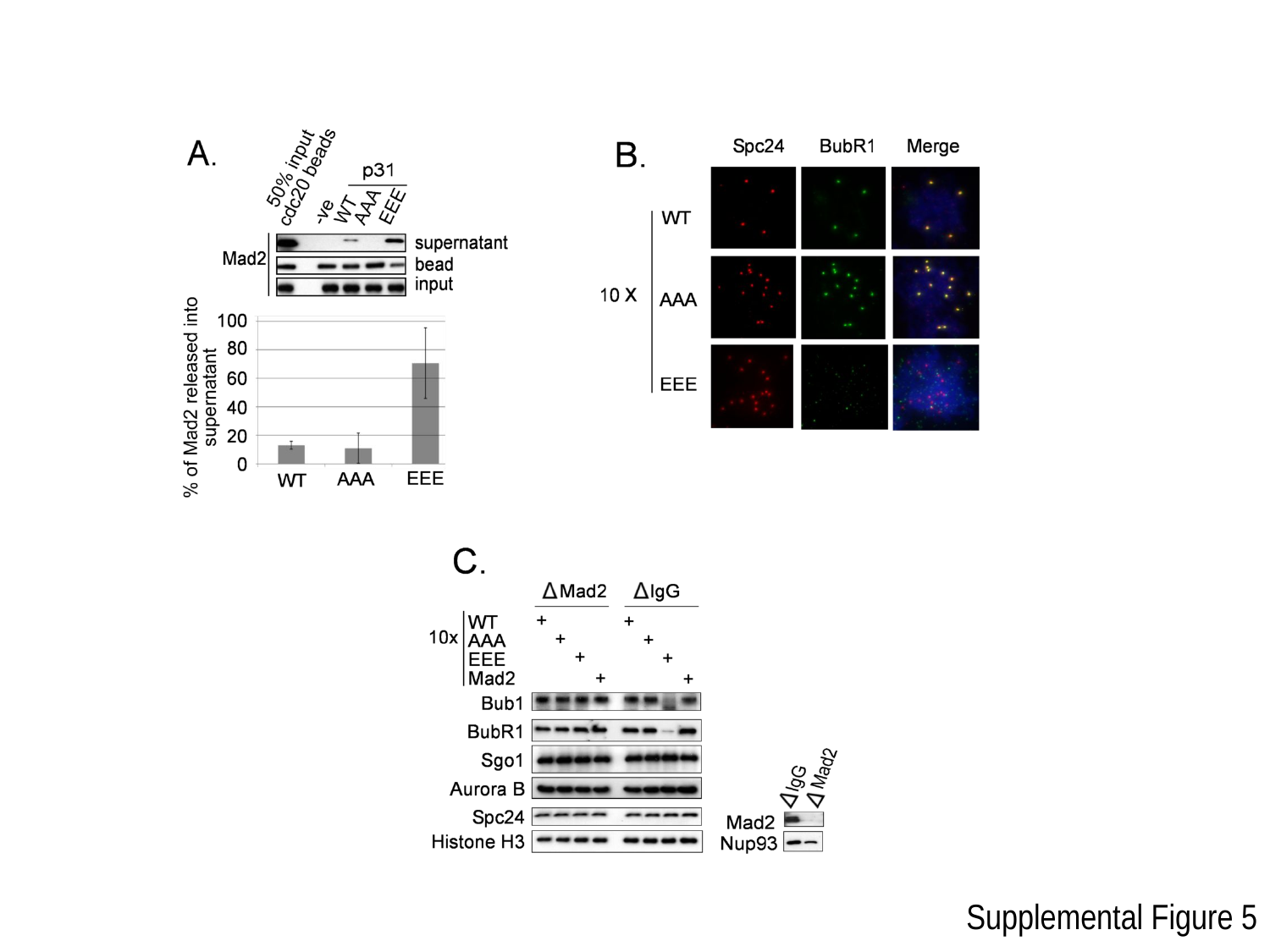

Supplemental Figure 5

## Slide 10
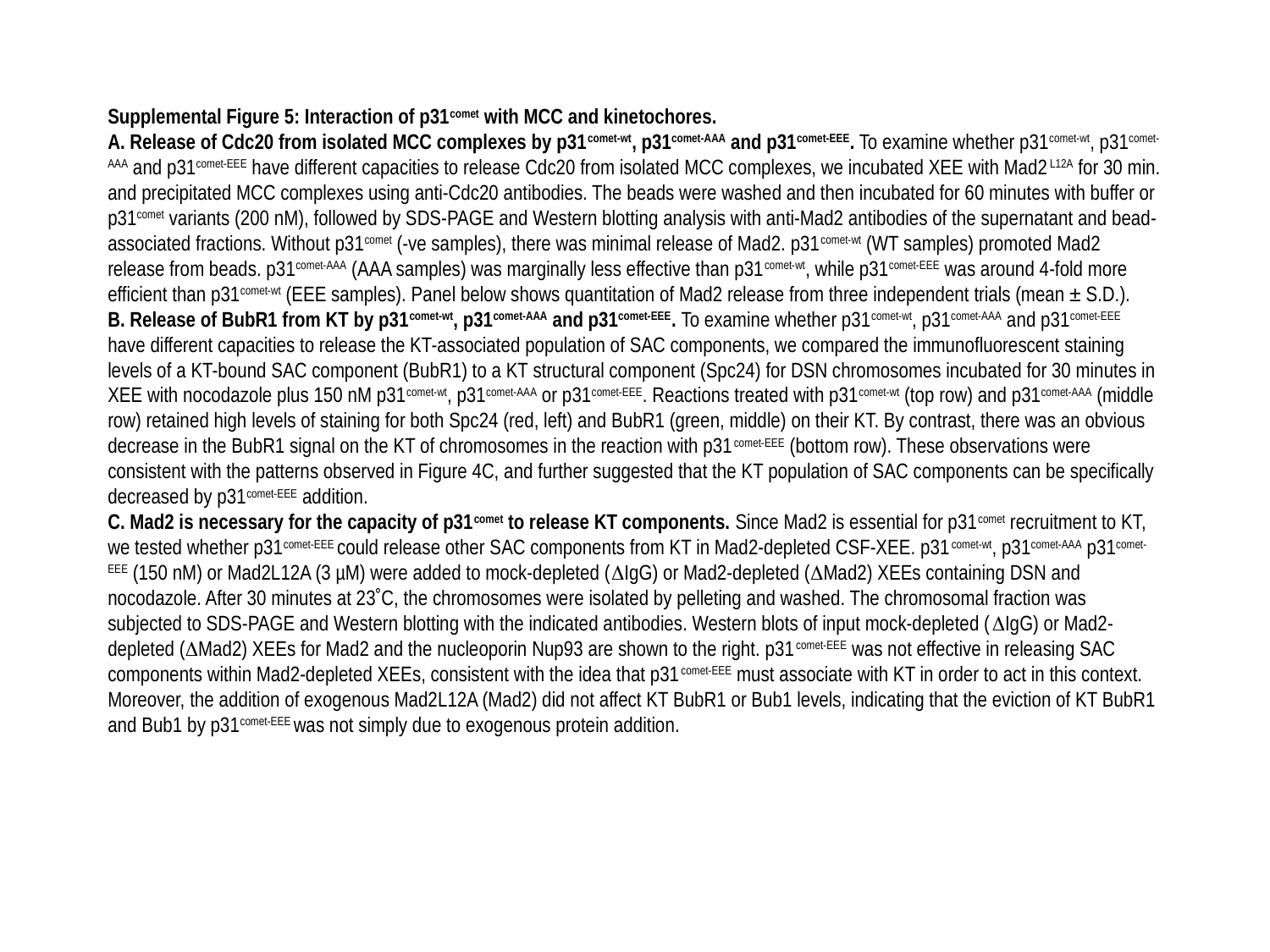

Supplemental Figure 5: Interaction of p31comet with MCC and kinetochores.
A. Release of Cdc20 from isolated MCC complexes by p31comet-wt, p31comet-AAA and p31comet-EEE. To examine whether p31comet-wt, p31comet-AAA and p31comet-EEE have different capacities to release Cdc20 from isolated MCC complexes, we incubated XEE with Mad2L12A for 30 min. and precipitated MCC complexes using anti-Cdc20 antibodies. The beads were washed and then incubated for 60 minutes with buffer or p31comet variants (200 nM), followed by SDS-PAGE and Western blotting analysis with anti-Mad2 antibodies of the supernatant and bead-associated fractions. Without p31comet (-ve samples), there was minimal release of Mad2. p31comet-wt (WT samples) promoted Mad2 release from beads. p31comet-AAA (AAA samples) was marginally less effective than p31comet-wt, while p31comet-EEE was around 4-fold more efficient than p31comet-wt (EEE samples). Panel below shows quantitation of Mad2 release from three independent trials (mean ± S.D.).
B. Release of BubR1 from KT by p31comet-wt, p31comet-AAA and p31comet-EEE. To examine whether p31comet-wt, p31comet-AAA and p31comet-EEE have different capacities to release the KT-associated population of SAC components, we compared the immunofluorescent staining levels of a KT-bound SAC component (BubR1) to a KT structural component (Spc24) for DSN chromosomes incubated for 30 minutes in XEE with nocodazole plus 150 nM p31comet-wt, p31comet-AAA or p31comet-EEE. Reactions treated with p31comet-wt (top row) and p31comet-AAA (middle row) retained high levels of staining for both Spc24 (red, left) and BubR1 (green, middle) on their KT. By contrast, there was an obvious decrease in the BubR1 signal on the KT of chromosomes in the reaction with p31comet-EEE (bottom row). These observations were consistent with the patterns observed in Figure 4C, and further suggested that the KT population of SAC components can be specifically decreased by p31comet-EEE addition.
C. Mad2 is necessary for the capacity of p31comet to release KT components. Since Mad2 is essential for p31comet recruitment to KT, we tested whether p31comet-EEE could release other SAC components from KT in Mad2-depleted CSF-XEE. p31comet-wt, p31comet-AAA p31comet-EEE (150 nM) or Mad2L12A (3 µM) were added to mock-depleted (ΔIgG) or Mad2-depleted (ΔMad2) XEEs containing DSN and nocodazole. After 30 minutes at 23˚C, the chromosomes were isolated by pelleting and washed. The chromosomal fraction was subjected to SDS-PAGE and Western blotting with the indicated antibodies. Western blots of input mock-depleted (ΔIgG) or Mad2-depleted (ΔMad2) XEEs for Mad2 and the nucleoporin Nup93 are shown to the right. p31comet-EEE was not effective in releasing SAC components within Mad2-depleted XEEs, consistent with the idea that p31comet-EEE must associate with KT in order to act in this context. Moreover, the addition of exogenous Mad2L12A (Mad2) did not affect KT BubR1 or Bub1 levels, indicating that the eviction of KT BubR1 and Bub1 by p31comet-EEE was not simply due to exogenous protein addition.

## Slide 11
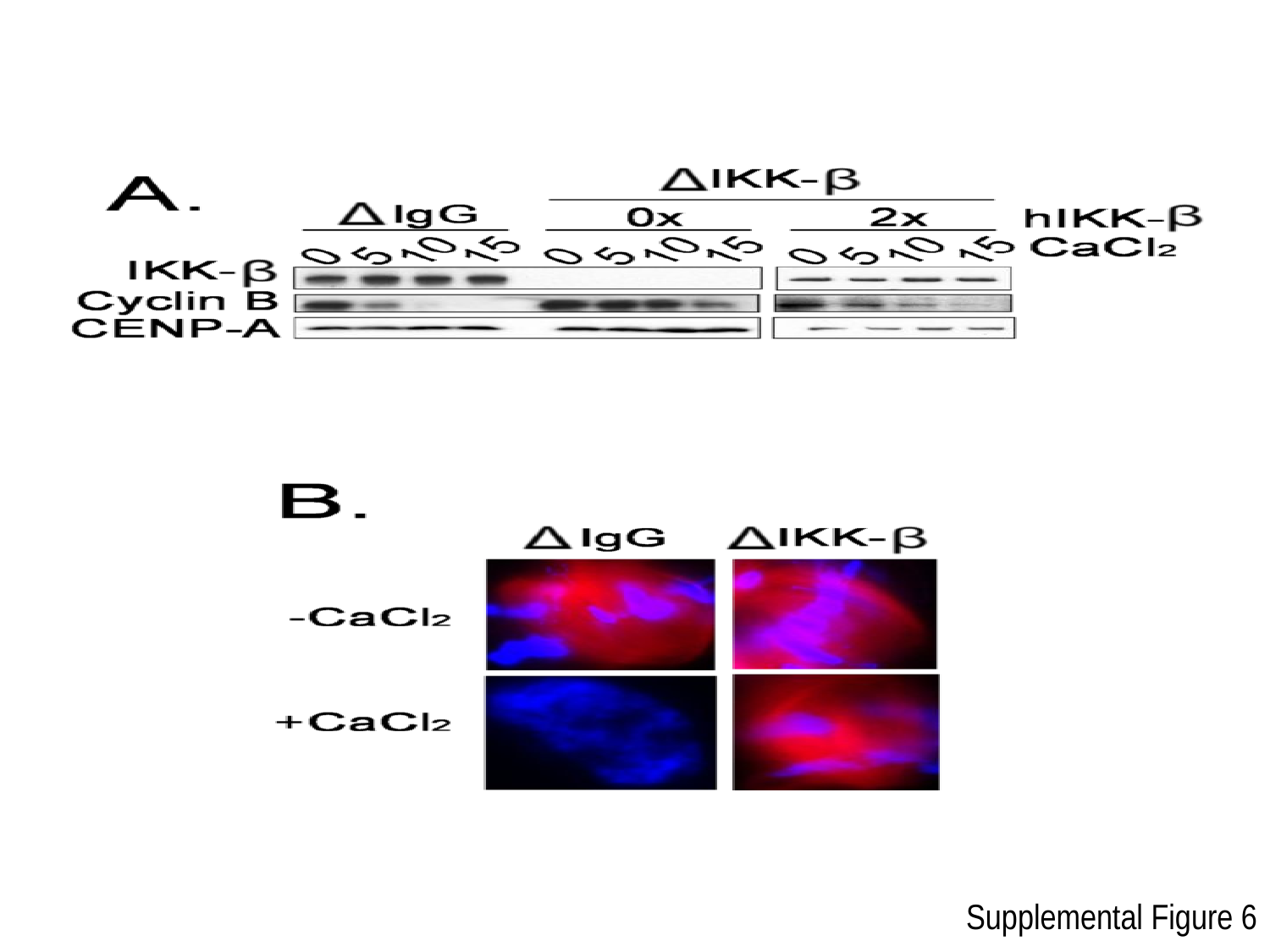

# Supplemental Figure 6

## Slide 12
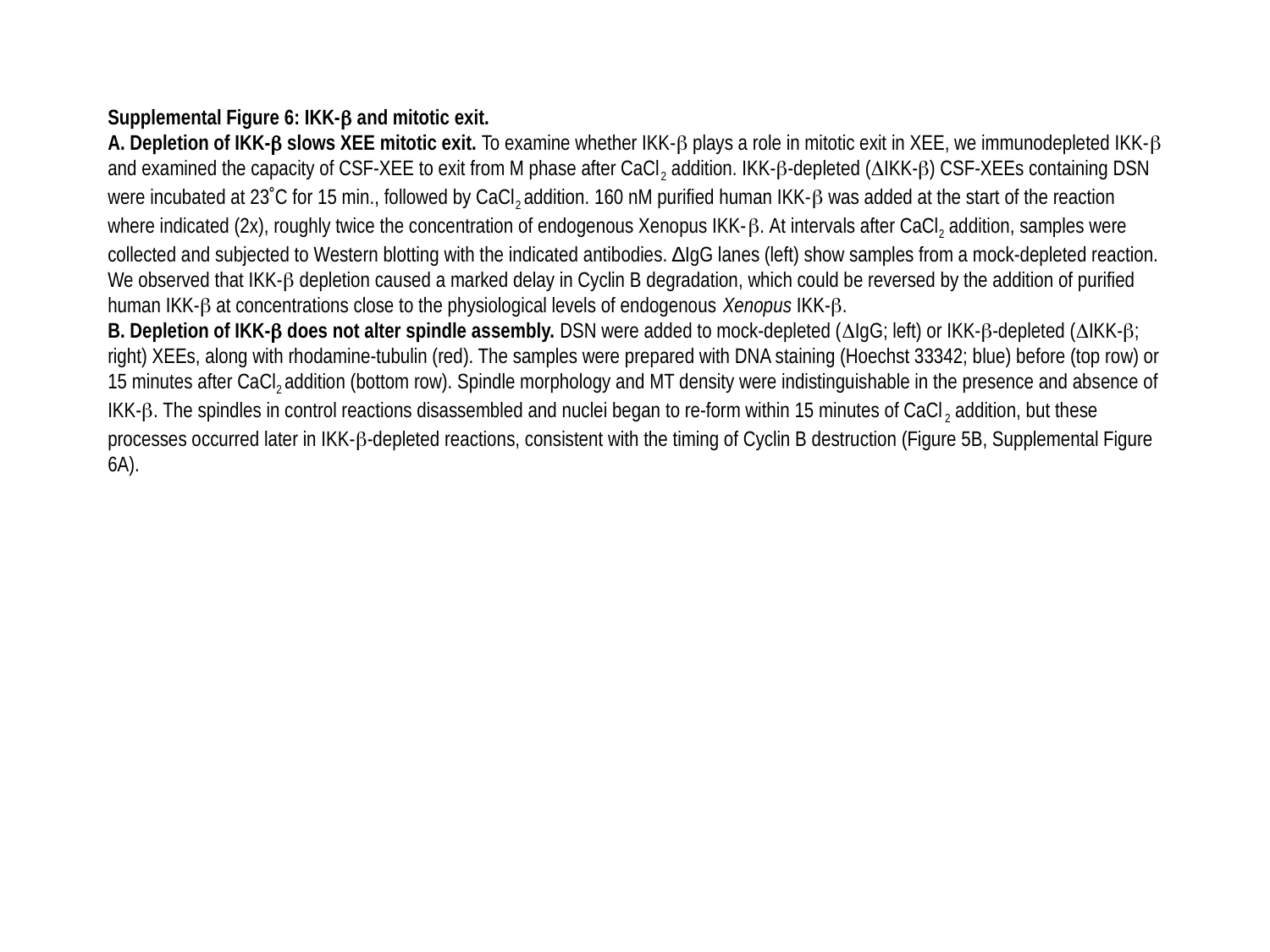

Supplemental Figure 6: IKK-β and mitotic exit.
A. Depletion of IKK-β slows XEE mitotic exit. To examine whether IKK-β plays a role in mitotic exit in XEE, we immunodepleted IKK-β and examined the capacity of CSF-XEE to exit from M phase after CaCl2 addition. IKK-β-depleted (ΔIKK-β) CSF-XEEs containing DSN were incubated at 23˚C for 15 min., followed by CaCl2 addition. 160 nM purified human IKK-β was added at the start of the reaction where indicated (2x), roughly twice the concentration of endogenous Xenopus IKK-β. At intervals after CaCl2 addition, samples were collected and subjected to Western blotting with the indicated antibodies. ∆IgG lanes (left) show samples from a mock-depleted reaction. We observed that IKK-β depletion caused a marked delay in Cyclin B degradation, which could be reversed by the addition of purified human IKK-β at concentrations close to the physiological levels of endogenous Xenopus IKK-β.
B. Depletion of IKK-β does not alter spindle assembly. DSN were added to mock-depleted (ΔIgG; left) or IKK-β-depleted (ΔIKK-β; right) XEEs, along with rhodamine-tubulin (red). The samples were prepared with DNA staining (Hoechst 33342; blue) before (top row) or 15 minutes after CaCl2 addition (bottom row). Spindle morphology and MT density were indistinguishable in the presence and absence of IKK-β. The spindles in control reactions disassembled and nuclei began to re-form within 15 minutes of CaCl2 addition, but these processes occurred later in IKK-β-depleted reactions, consistent with the timing of Cyclin B destruction (Figure 5B, Supplemental Figure 6A).

## Slide 13
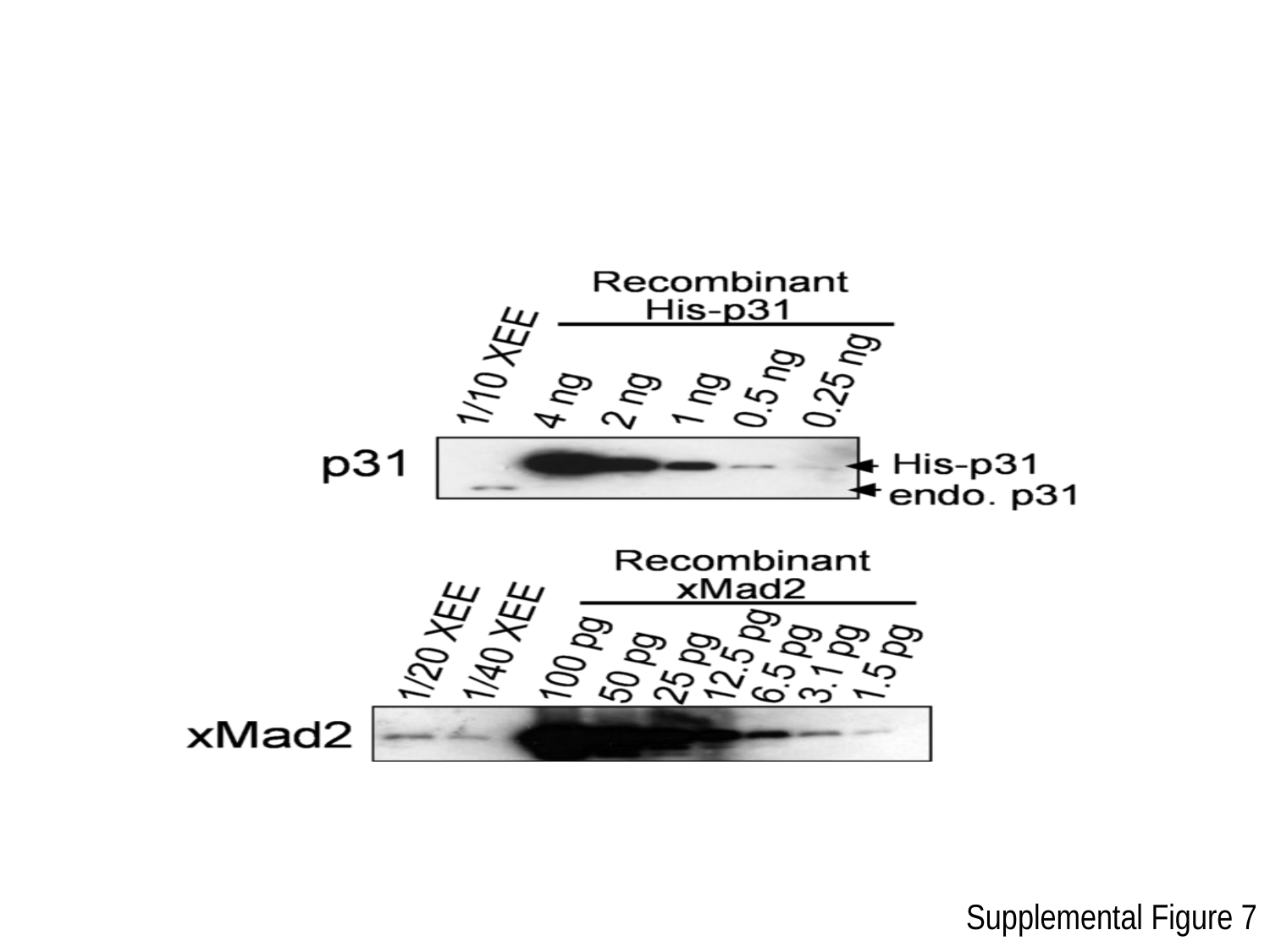

# Supplemental Figure 7

## Slide 14
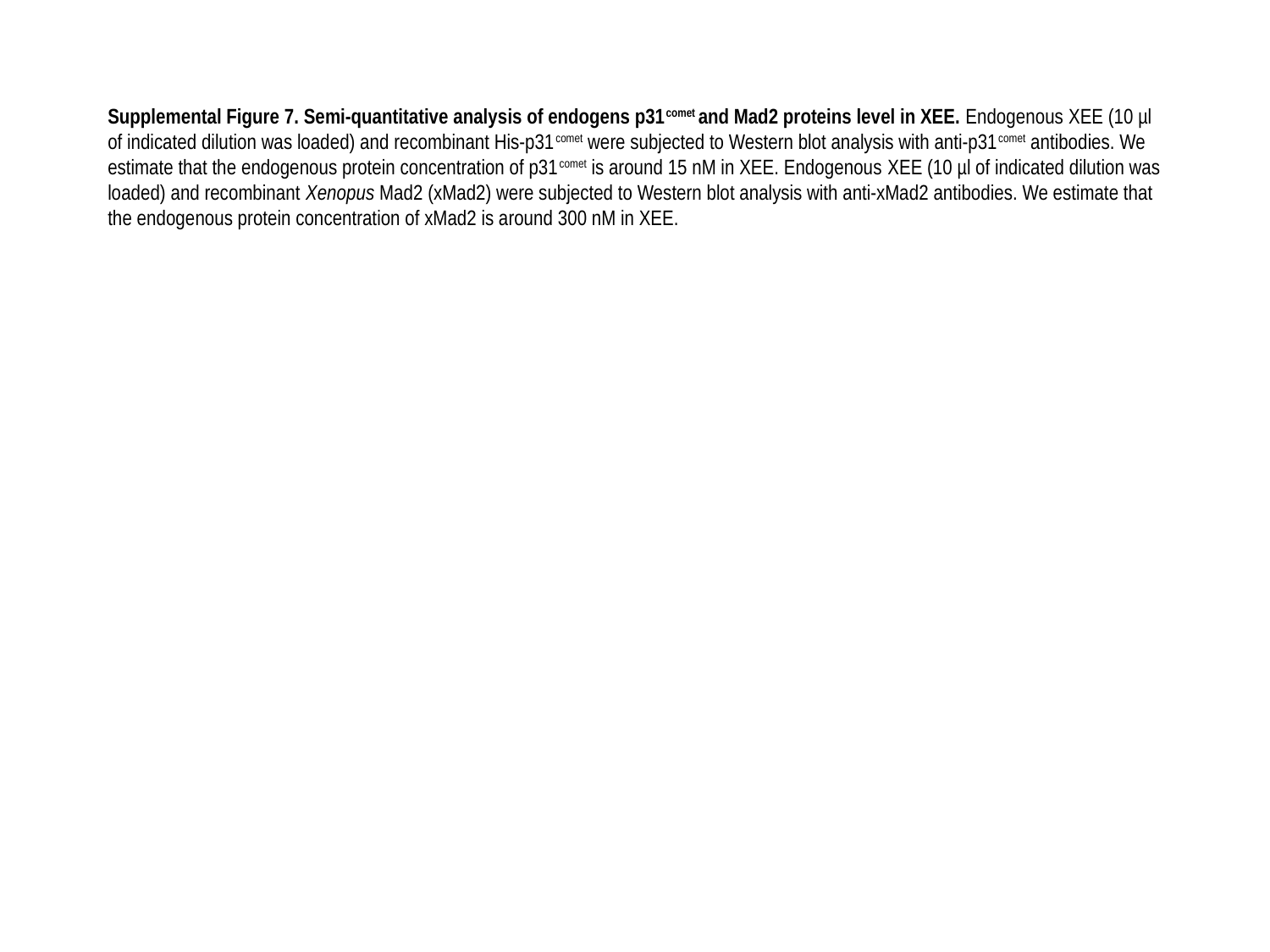

Supplemental Figure 7. Semi-quantitative analysis of endogens p31comet and Mad2 proteins level in XEE. Endogenous XEE (10 µl of indicated dilution was loaded) and recombinant His-p31comet were subjected to Western blot analysis with anti-p31comet antibodies. We estimate that the endogenous protein concentration of p31comet is around 15 nM in XEE. Endogenous XEE (10 µl of indicated dilution was loaded) and recombinant Xenopus Mad2 (xMad2) were subjected to Western blot analysis with anti-xMad2 antibodies. We estimate that the endogenous protein concentration of xMad2 is around 300 nM in XEE.

## Slide 15
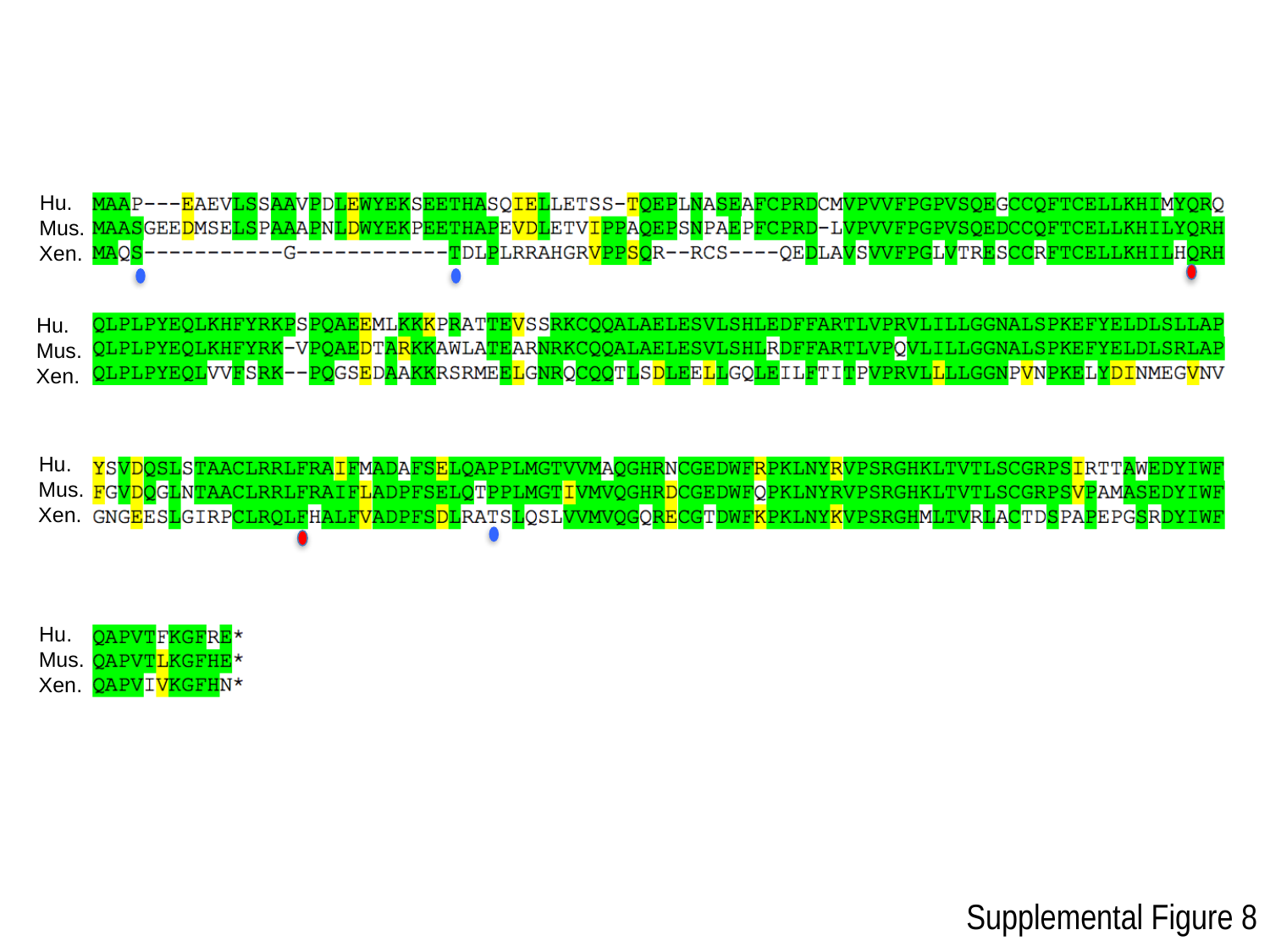

Hu.
Mus.
Xen.
Hu.
Mus.
Xen.
Hu.
Mus.
Xen.
Hu.
Mus.
Xen.
 Supplemental Figure 8

## Slide 16
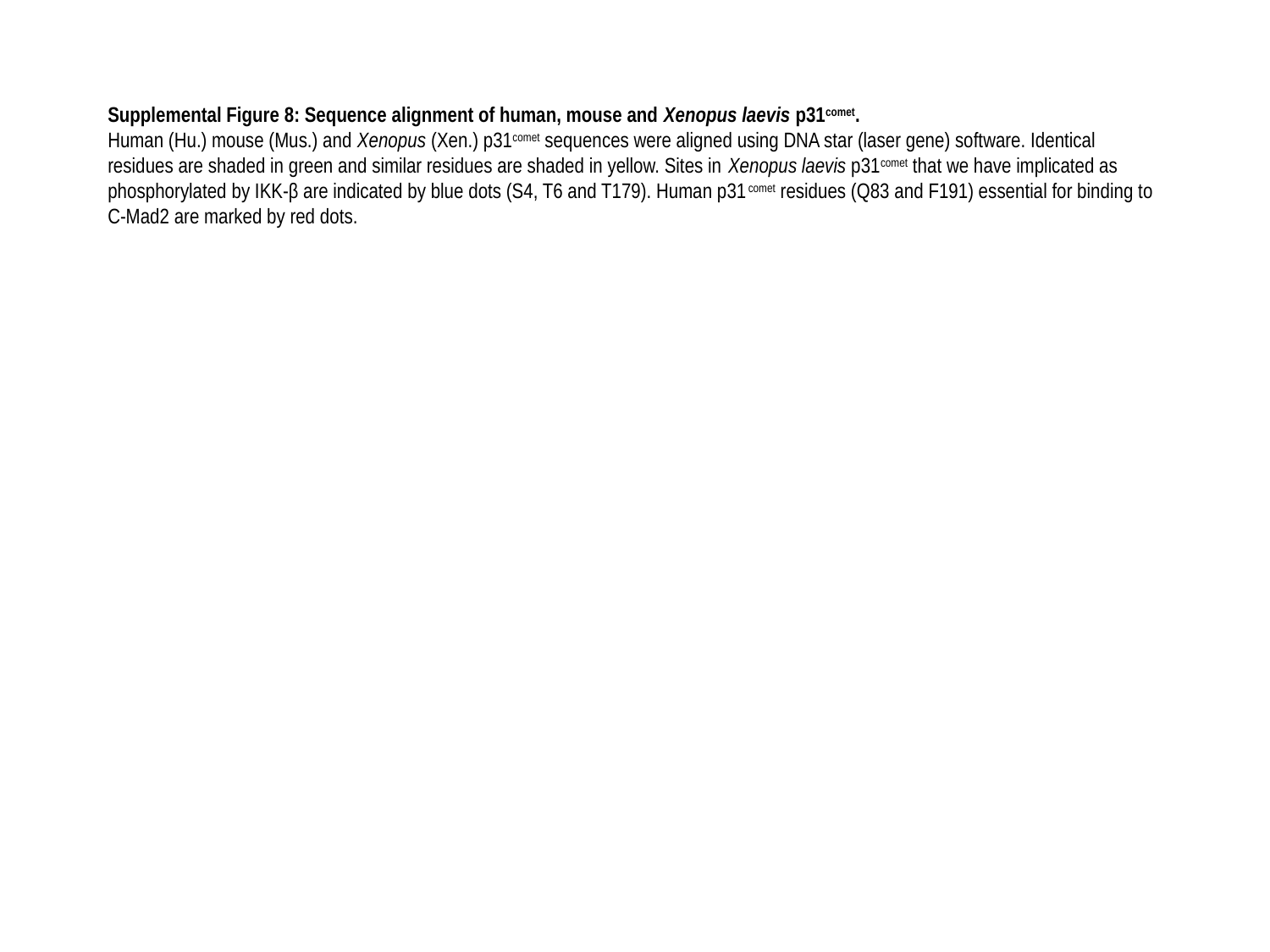

Supplemental Figure 8: Sequence alignment of human, mouse and Xenopus laevis p31comet.
Human (Hu.) mouse (Mus.) and Xenopus (Xen.) p31comet sequences were aligned using DNA star (laser gene) software. Identical residues are shaded in green and similar residues are shaded in yellow. Sites in Xenopus laevis p31comet that we have implicated as phosphorylated by IKK-β are indicated by blue dots (S4, T6 and T179). Human p31comet residues (Q83 and F191) essential for binding to C-Mad2 are marked by red dots.
